# Comparative effectiveness of antihypertensive medication for primary prevention of cardiovascular disease: systematic review and multiple treatments meta-analysis

**DOI:** 10.1186/1741-7015-10-33

**Published:** 2012-04-05

**Authors:** Atle Fretheim, Jan Odgaard-Jensen, Odd Brørs, Steinar Madsen, Inger Njølstad, Ole F Norheim, Arne Svilaas, Ivar S Kristiansen, Hanne Thürmer, Signe Flottorp

**Affiliations:** 1 Global Health Unit, Norwegian Knowledge Centre for the Health Services, Oslo, Norway; 2 Institute of Health and Society, University of Oslo, Oslo, Norway; 3 Department of Clinical Pharmacology, Oslo University Hospital, Oslo, Norway; 4 Department of Medical Information, Norwegian Medicines Agency, Oslo, Norway; 5 Department of Community Medicine, University of Tromsø, Tromsø, Norway; 6 Department of Medicine, Haukeland University Hospital, Bergen, Norway; 7 Department of Public Health and Primary Health Care, University of Bergen, Bergen, Norway; 8 Lipid Clinic, Oslo University Hospital, Oslo, Norway; 9 Medical Department Notodden, Telemark Hospital, Notodden, Norway; 10 Prevention, Health Promotion and Organisation Unit, Norwegian Knowledge Centre for the Health Services, Oslo, Norway

## Abstract

**Background:**

We conducted a systematic review of evidence from randomized controlled trials to answer the following research question: What are the relative effects of different classes of antihypertensive drugs in reducing the incidence of cardiovascular disease outcomes for healthy people at risk of cardiovascular disease?

**Methods:**

We searched MEDLINE, EMBASE, AMED (up to February 2011) and CENTRAL (up to May 2009), and reference lists in recent systematic reviews. Titles and abstracts were assessed for relevance and those potentially fulfilling our inclusion criteria were then assessed in full text. Two reviewers made independent assessments at each step. We selected the following main outcomes: total mortality, myocardial infarction and stroke. We also report on angina, heart failure and incidence of diabetes. We conducted a multiple treatments meta-analysis using random-effects models. We assessed the quality of the evidence using the GRADE-instrument.

**Results:**

We included 25 trials. Overall, the results were mixed, with few significant dif-ferences, and with no drug-class standing out as superior across multiple outcomes. The only significant finding for total mortality based on moderate to high quality evidence was that beta-blockers (atenolol) were inferior to angiotensin receptor blockers (ARB) (relative risk (RR) 1.14; 95% credibility interval (CrI) 1.02 to 1.28). Angiotensin converting enzyme (ACE)-inhibitors came out inferior to calcium-channel blockers (CCB) regarding stroke-risk (RR 1.19; 1.03 to 1.38), but superior regarding risk of heart failure (RR 0.82; 0.69 to 0.94), both based on moderate quality evidence. Diuretics reduced the risk of myocardial infarction compared to beta-blockers (RR 0.82; 0.68 to 0.98), and lowered the risk of heart failure compared to CCB (RR 0.73; 0.62 to 0.84), beta-blockers (RR 0.73; 0.54 to 0.96), and alpha-blockers (RR 0.51; 0.40 to 0.64). The risk of diabetes increased with diuretics compared to ACE-inhibitors (RR 1.43; 1.12 to 1.83) and CCB (RR 1.27; 1.05 to 1.57).

**Conclusion:**

Based on the available evidence, there seems to be little or no difference between commonly used blood pressure lowering medications for primary prevention of cardiovascular disease. Beta-blockers (atenolol) and alpha-blockers may not be first-choice drugs as they were the only drug-classes that were not significantly superior to any other, for any outcomes.

Review registration: CRD database ("PROSPERO") CRD42011001066

## Background

### Rationale

Cardiovascular diseases are a major public health challenge, representing 10% of the global burden of disease [1]. The annual number of deaths caused by cardiovascular disease is expected to rise by more than 33% over the coming two or three decades [2]. Hypertension is among the most important modifiable risk-factors for cardiovascular diseases [3]. Meta-analyses of placebo-controlled trials of antihypertensive medication have shown that such treatment can prevent, or postpone myocardial infarction and stroke [4]. But the key question remains: Which of the many available types of blood pressure lowering drugs is the better choice as first-line medication?

Several clinical trials and systematic reviews have addressed this issue, but have failed to convincingly show that one or more drug-classes are superior to the others [5-9]. Still, controversy remains about possible important differences between the various drugs. The findings from the alpha-blocker arm of the ALLHAT-trial a decade ago [10], and reviews in recent years assessing the effectiveness of beta-blocking agents [11,12] cast doubt about the assumption that all antihypertensive drugs are equally effective with regards to cardiovascular disease prevention. Also, recent systematic reviews have found potentially important differences regarding their effectiveness for some specific outcomes [13,14].

Systematic reviews of randomized controlled trials comparing different drugs provide evidence for decisions about choice of antihypertensive medication. Unfortunately, direct comparative studies are lacking for many of the competing drug-classes. Conventional meta-analyses of antihypertensive medication, therefore, typically provide comparative effectiveness estimates for only some drug-comparisons, that is, those that have been tested head-to-head in clinical trials. However, a decision maker would want to have effect-estimates for as many comparisons as possible, preferably with a ranking of the various drugs. Multiple treatments (network) meta-analyses provide this by utilizing indirect comparisons, making it possible to estimate the comparative effectiveness of drugs that have not been tested directly in clinical trials [8,13,14].

The most recent systematic review addressing several of the most clinically important outcomes and using multiple treatments meta-analysis of antihypertensive drug therapy was published by Psaty and colleagues in 2003 [8]. An update is warranted to reflect the current evidence-base in the field and to address some shortcomings of the earlier review, for example, that the authors neither explicitly assessed the risk of bias in the included studies, nor graded the quality of the overall body of evidence.

A broad systematic review of various interventions for primary prevention of cardiovascular diseases was recently requested by the Norwegian Directorate for Health [15,16]. The current paper is an updated and substantially revised version of that report's section on antihypertensive treatment.

### Objectives

Our study was designed to answer the following research question: What are the relative effects of different classes of antihypertensive drugs in reducing the incidence of cardiovascular disease outcomes for healthy people at risk of cardiovascular disease?

## Methods

### Protocol

Methods for this review were specified in advance, and registered in the PROSPERO-database [17].

### Eligibility criteria

We only included randomized controlled trials comparing one or more drugs against each other, or no active treatment. Since our focus was on primary prevention, that is, the participants should be free from cardiovascular disease, we pragmatically excluded trials where more than half the participants had had a myocardial infarction, stroke or other significant cardiovascular event. We also excluded studies done exclusively in selected subgroups of patients with hypertension, for example, patients with diabetes or microalbuminuria.

Only trials of drugs belonging to commonly used "drug-classes" were included: Diuretics, beta-blockers, calcium-channel blockers (CCB), angiotensin converting enzyme inhibitors (ACE-inhibitors), angiotensin receptor blockers (ARB), and alpha-blockers. Because we were only interested in studies of "low-dose" diuretics, we excluded trials where the maximum dosage in the treatment protocol was higher than the "optimal range" as proposed by the Joint National Committee U.S. guidelines [18] and the authors of a recent Cochrane review [19].

To be considered for inclusion, a trial had to have cardiovascular morbidity or mortality as a primary outcome, either explicitly stated by the authors or based on our judgement. In practice, this meant excluding many smaller studies, typically designed to evaluate effects on surrogate outcomes such as blood pressure. Some of these trials reported morbidity and mortality outcomes in addition to their primary surrogate outcome. We disregarded these data in the belief that the information was not likely to be important given the availability of findings from large-scale studies with morbidity and mortality as the main outcomes. By doing this, we also reduced the risk of introducing certain biases in our analyses, for example, due to selective reporting of findings, which is likely to be a greater problem with smaller studies [20].

### Information sources and search

We searched MEDLINE, EMBASE, AMED (up to February 2011) and CENTRAL (up to May 2009). See Additional file 1 for the complete search strategy. We also studied the reference lists in recent systematic reviews. We did not apply any language re-strictions.

### Study selection

We first assessed the relevance of titles and abstracts that the search yielded. Secondly, potentially relevant articles were assessed in full text. At each step two reviewers made independent assessments.

### Data collection process

Data extraction was done independently by two reviewers using a simple, standardized form.

### Data items

We extracted, where possible, data for the following main outcomes from all the included studies: total mortality, myocardial infarction and stroke. In addition, we extracted data on the following outcomes: angina, heart failure and diabetes.

### Risk of bias in individual studies

Studies fulfilling our eligibility criteria were assessed for internal validity at the study level by two reviewers independently using a standard check list [21]. Studies were excluded if the validity was judged as "low".

At each step disagreements between two reviewers' assessments were resolved through discussion, for example, through e-mail discussions or at plenary meetings for the whole group of reviewers.

### Summary measures

We expressed the comparative effectiveness of the treatments as the relative risk (RR) of an outcome, with 95% credibility intervals (CrIs). The credibility interval is the Bayesian analogue to confidence intervals used in traditional frequentist statistical approaches. We considered a result "significant" if the CrI did not include RR = 1.

We also ranked the different drug-classes in terms of their likelihood of leading to the best results for each outcome. We chose to report on the probability that a drug-class would be among the three best drugs for each outcome.

### Synthesis of results

The analysis was primarily based on Multiple Treatments Meta-analysis (MTM) as described by Salanti [22]. We used the arm-based network meta-analyses method (a Bayesian method based on Markov Chain Monte Carlo simulation) [22]. All MTM were performed using Winbugs version 1.4.3 (Imperial College and MRC, UK). We utilized random effects models. The statistical analysis is based on binomial likelihoods, with vague priors for the trial baselines, basic parameters (normal distribution with mean 0 and standard deviation 0.0001) and the random effects standard deviation (uniformly distributed i in the interval 0 to 2), and takes the correlation structure induced by multi-arm trials into account. We have used a random effects model which follows the Normal distribution with 0 as the mean.

We checked for inconsistency between direct and indirect evidence by "node-splitting" [23]. We calculated the direct and indirect estimates of effect and the corresponding Bayesian "*P*-values" for inconsistency.

### Quality-assessment of the evidence

We assessed the quality of the evidence for each outcome for each comparison, using the GRADE-instrument (Grading of Recommendations Assessment, Development and Evaluation - GRADE) [24]. Through this process, factors like study limitations, inconsistency of results, indirectness of evidence, imprecision and reporting bias are assessed. The quality of the evidence was rated as high, moderate, low or very low quality, reflecting the confidence we have in the estimated effect size (see Table 1).

**Table 1:** Grades of quality of evidence in GRADE [24]

**High **= We are very confident that the true effect lies close to that of the estimate of the effect.
**Moderate **= We are moderately confident in the effect estimate: The true effect is likely to be close to the estimate of the effect, but there is a possibility that it is substantially different.

**Low **= Our confidence in the effect estimate is limited: The true effect may be substantially different from the estimate of the effect.

**Very low **= We have very little confidence in the effect estimate: The true effect is likely to be substantially different from the estimate of effect.

In line with recommendations from the GRADE Working Group by default, we graded the included evidence as "high quality", as all studies were randomized controlled trials. We then downgraded when deemed appropriate. For the comparisons where we had no direct evidence (that is, the effect-estimates were only based on indirect comparisons) we rated the quality as "low" unless we found reasons to upgrade or to downgrade further. When the findings were based on a combination of direct and indirect evidence we elected to grade as "high quality" unless there were reasons to downgrade. The inconsistency of results-dimension in GRADE was only assessed for direct comparisons (using the I^2^-statistic). The grading process was done by one reviewer (AF) and validated by a second person.

## Results

### Study selection

**Figure 1:**
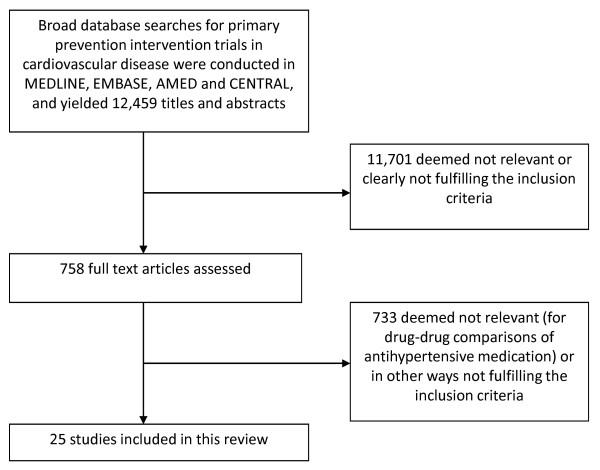
**Review flowchart**.

Our search yielded 12,499 references, of which 758 were deemed as potentially meeting our inclusion criteria, and the complete articles studied. Among these, 25 trials of antihypertensive medications met the inclusion criteria and were of sufficient quality to be included in our systematic review (Figure 1). A list of excluded studies is found in Additional file 2.

### Study characteristics

Key characteristics of included studies are presented in Table 2 [10,25-49]. Atenolol was used in all the trials with beta-blockers.

**Table 2:** Overview of included trials

Study, year (reference)	Comparisons (initial drug-dosages)	Sample size (total, included in analysis)	Age group (years)	Follow-up (mean, years)
**VA II 1970 **[43]	Hydrochlorothiazide (50 mg/day) + reserpine (0.1 mg/day) + hydralazine hydrochloride (25 mg/day) vs. placebo	380	30 to 73	3.3

**Oslo hypertension study 1980 **[27]	Hydrochlorothiazide (50 mg/day) vs. no treatment	785	40 to 49	5.5

**EWPHE 1985 **[44]	Hydrochlorothiazide (25 mg/day) + triamteren (50 mg/day) vs. placebo	840	> 60	4.7

**Coope 1986 **[45]	Atenolol (100 mg/day) or bendrofluazide (5 mg/day) vs. no treatment	884	60 to 79	4.4

**HAPPHY 1987 **[28]	Bendroflumethiazide (5 mg/day) or hydrochlorothiazide (50 mg/day) vs. atenolol (100 mg/day) or metoprolol 200 mg/day)	6,569	40 to 64	3.8

**STOP 1 1991 **[46]	Atenolol (50 mg/day) or HCTZ (25 mg/day) (add-on drugs: amiloride (2.5 mg/day) or metoprolol (100 mg/day) or pindolol (5 mg/day)) vs. placebo	1,627	70 to 84	2

**SHEP 1991 **[26]	Chlortalidone (12.5 mg/day) vs. placebo	4,736	> 60	4.5

**MRC 2 1992 **[29]	Hydrochlorothiazide+amiloride (25 + 2.5 mg/day) vs. atenolol (50 mg/day) vs. placebo	4,396	65 to 74	5.8

**SYST-EUR 1997 **[47]	Nitrendipine (10 mg/day) vs. placebo	4,695	> 60	4

**Sun 1997 **[25]	Nitrendipine (10 mg/day) (starting dose: 10 mg × 3, reduced to 10 mg × 1) vs. usual care	2,080	> 15	4.7

**CAPPP 1999 **[30]	Captopril (50 mg/day) vs. atenolol (50 to 100 mg/day) or metoprolol (50 to 100 mg/day) or hydrochlorothiazide (25 mg/day) or bendroflumethiazide (2.5 mg/day)	10,985	25 to 66	6.1

**NICS-EH 1999 **[31]	Nicardipine (20 mg × 2/day) vs. Trichlormethiazide (2 mg/day)	414	≥ 60	4.6 (nicardipine group) and 3.9 (trichlormethia-zide group)

**STOP-2 1999 **[32]	Atenolol (50 mg/day) or metoprolol (100 mg/day) or pindolol (5 mg/day) or hydrochlorothiazide (25 mg/day)+amilorid (2.5 mg/day) vs. enalapril (10 mg/day) or lisinopril (10 mg/day) vs. felodipine (2.5 mg/day) or isradipine (2.5 mg/day)	6,614	70 to 84	4 to 6

**ALLHAT 2000, 2002 **[10,33]	Doxazosine (2 mg/day) vs. chlorthalidone (12.5 mg/day) vs. amlodipine (2.5 mg/day) vs. lisinopril (10 mg/day)	42,424	> 55	4.9 for chlorthalidone vs. amlodipine vs. lisinopril. 3.3 (median) for chlorthalidone vs. doxazosine

**NORDIL 2000 **[34]	Diltiazem (180 to 360 mg/day) vs. thiazide (not specified) or beta-blocker (not specified)	10,881	50 to 74	4.5

**INSIGHT 2000 **[35]	Nifedipine (30 mg/day) vs. hydrochlorothiazide (25 mg/day)+amilorid (2.5 mg/day)	6,321	55 to 80	~3.5

**LIFE 2002 **[36]	Losartan (50 mg/day) vs. atenolol (50 mg/day)	9,193	55 to 80	4.8

**ANBP 2 2003 **[37]	ACE-inhibitor (unspecified type and dosage) vs. thiazide-diuretic (unspecified type and dosage)	6,083	65 to 84	4.1

**CONVINCE 2003 **[38]	Verapamil (180 mg/day) vs. atenolol (50 mg/day) or hydrochlorothiazide (12.5 mg/day)	16,602	> 55	3

**HYVET-PILOT 2003 **[39]	Lisinopril (2.5 mg/day) vs. bendroflumethiazide (2.5 mg/day) vs. no treatment	857	> 80	1.1

**SHELL 2003 **[40]	Chlorthalidone (12.5 mg/day) vs. lacidipine (4 mg/day)	1,882	> 60	2.7 (median)

**VALUE 2004 **[49]	Valsartan (80 mg/day) vs. amlodipine (5 mg/day)	15,245	> 50	4.2

**E-COST 2005 **[41]	Candesartan (4 or 8 mg/day) vs. "conventional based regimen"	1,630	35 to 79	3.1

**CASE-J 2008 **[42]	Candesartan (8 mg/day) vs. amlodipine (5 mg/day)	4,703	63.8 (mean)	3.2

**HYVET 2008 **[48]	Indapamid (1.5 mg/day) vs. placebo	3,845	≥ 80	2.1

Eleven of the identified studies only partially addressed our research question [30,32,34,38,41,50-55]. Four of these trials were not direct comparisons between individual drug-classes, but rather comparisons of one drug against a diuretic or a beta-blocker, selected at the physician's discretion [30,32,34,38]. We decided to include these studies by defining "diuretics or beta-blockers" as a separate drug-class in our analyses. Similarly, one trial compared ARB and "conventional treatment" [41], which we also included as a separate drug-class. Three of the trials were comparisons between different drug combinations or add-on drugs, and we decided to exclude these [51-53]. Finally, we excluded three placebo-controlled trials where the investigators aimed at achieving similar blood pressure levels in both groups since in these trials a large proportion in the placebo-group also received active treatment, making it difficult to interpret the findings for our purpose [50,54,55].

For three of the included studies [10,33,49,51], we were in doubt whether to include or exclude, due to the high proportion of participants with pre-existing cardiovascular disease. In these trials, this proportion was probably slightly above our pragmatically selected cut-off point of 50%. However, as we had not clearly defined "cardiovascular event" and since the exact proportion of participants with established cardiovascular disease was difficult to discern from the study reports, we decided to include these studies.

### Synthesis of results

#### Relative risk estimates and quality of evidence

**Figure 2:**
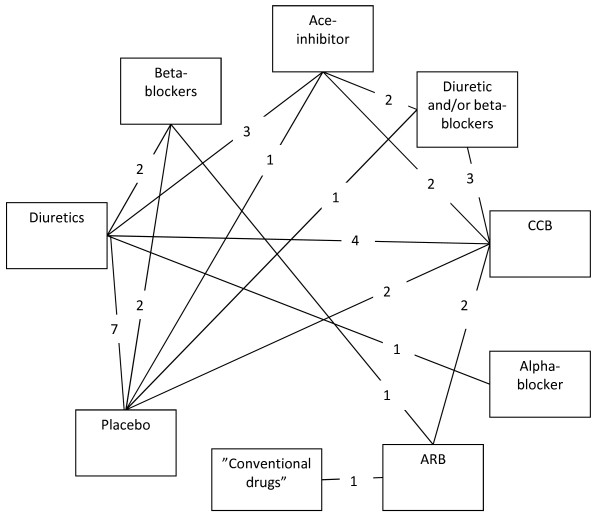
**Direct comparisons in network model**.

The various drug-classes and number of studies per direct comparison included in our network-analysis are shown in Figure 2.

Tables 3 and 4 display effect estimates for our primary and secondary outcomes, respectively. The tables also include the results of the quality assessment of the evidence underlying each effect estimate. We have limited the tables to include only the most clinically relevant comparisons, that is, we have excluded "diuretics and/or beta-blockers", "conventional drugs" or "placebo/control". The complete presentation is found in Additional files 3 and 4. A detailed presentation of the quality of evidence assessments is found in Additional file 5.

**Table 3:** Main findings (primary outcomes).

	All-cause mortality	Myocardial infarction	Stroke
Diuretics vs.BB	0.90 (0.80 to 1.01)	0.82 (0.68 to 0.98)	0.83 (0.68 to 1.07)
	
	⊕ ⊕ ⊕ O	⊕ ⊕ OO	⊕ ⊕ OO

Diuretics vs. ACE	1.00 (0.93 to 1.08)	1.00 (0.88 to 1.15)	0.94 (0.81 to 1.10)
	
	⊕ ⊕ ⊕ O	⊕ ⊕ ⊕ O	⊕ ⊕ ⊕ O

Diuretics vs. CCB	1.03 (0.96 to 1.10)	0.96 (0.84 to 1.07)	1.12 (0.97 to 1.29)
	
	⊕ ⊕ ⊕ O	⊕ ⊕ ⊕ O	⊕ ⊕ OO

Diuretics vs.alpha-blockers	0.98 (0.87 to 1.12)	0.99 (0.80 to 1.23)	0.85 (0.66 to 1.12)
	
	⊕ ⊕ ⊕ O	⊕ ⊕ ⊕ O	⊕ ⊕ OO

Diuretics vs.ARB	1.02 (0.92 to 1.14)	0.83 (0.69 to 1.03)	1.02 (0.82 to 1.28)
	
	⊕ ⊕ OO	⊕ OOO	⊕ OOO

BB vs. ACE	1.12 (0.98 to 1.27)	1.22 (1.00 to 1.52)	1.13 (0.86 to 1.42)
	
	⊕ OOO	⊕ ⊕ OO	⊕ OOO

BB vs. CCB	1.14 (1.01 to 1.28)	1.17 (0.97 to 1.42)	1.34 (1.05 to 1.64)
	
	⊕ ⊕ OO	⊕ OOO	⊕ ⊕ OO

BB vs. alpha-blockers	1.09 (0.93 to 1.30)	1.20 (0.92 to 1.61)	1.02 (0.71 to 1.42)
	
	⊕ OOO	⊕ OOO	⊕ OOO

BB vs. ARB	1.14 (1.02 to 1.28)	1.02 (0.84 to 1.27)	1.23 (0.96 to 1.49)
	
	⊕ ⊕ ⊕ ⊕	⊕ ⊕ ⊕ O	⊕ ⊕ ⊕ O

ACE vs. CCB	1.02 (0.95 to 1.10)	0.96 (0.83 to 1.07)	1.19 (1.03 to 1.38)
	
	⊕ ⊕ ⊕ O	⊕ ⊕ ⊕ O	⊕ ⊕ ⊕ O

ACE vs. alpha-blockers	0.98 (0.85 to 1.14)	0.99 (0.77 to 1.27)	0.91 (0.67 to 1.24)
	
	⊕ ⊕ OO	⊕ OOO	⊕ OOO

ACE vs. ARB	1.02 (0.91 to 1.14)	0.84 (0.68 to 1.04)	1.08 (0.86 to 1.37)
	
	⊕ ⊕ OO	⊕ OOO	⊕ OOO

CCB vs. alpha-blockers	0.96 (0.83 to 1.11)	1.03 (0.82 to 1.34)	0.77 (0.57 to 1.04)
	
	⊕ ⊕ OO	⊕ OOO	⊕ OOO

CCB vs. ARB	1.00 (0.91 to 1.10)	0.87 (0.74 to 1.06)	0.91 (0.75 to 1.11)
	
	⊕ ⊕ ⊕ ⊕	⊕ ⊕ ⊕ O	⊕ ⊕ ⊕ O

Alpha-blockers vs. ARB	1.04 (0.88 to 1.23)	0.84 (0.63 to 1.14)	1.20 (0.85 to 1.69)
	
	⊕ ⊕ OO	⊕ OOO	⊕ OOO

ACE, Angiotensin Converting Enzyme Inhibitors; ARB, Angiotensin Receptor Blockers; BB, Beta-blockers; CCB, Calcium Channel Blockers; ⊕ ⊕ ⊕ ⊕, High quality evidence; ⊕ ⊕ ⊕ O, Moderate quality evidence; ⊕ ⊕ OO, Low quality evidence; ⊕ OOO, Very low quality evidence

**Table 4:** Main findings (secondary outcomes).

	Angina	Heart failure	Diabetes incidence
Diuretics vs. BB	0.96 (0.28 to 5.78)	0.73 (0.54 to 0.96)	1.09 (0.80 to 1.44)
	
	⊕ OOO	⊕ ⊕ ⊕ O	⊕ ⊕ OO

Diuretics vs. ACE	0.97 (0.42 to 2.51)	0.88 (0.76 to 1.06)	1.43 (1.12 to 1.83)
	
	⊕ ⊕ OO	⊕ ⊕ OO	⊕ ⊕ ⊕ O

Diuretics vs. CCB	1.05 (0.56 to 2.19)	0.73 (0.62 to 0.84)	1.27 (1.05 to 1.57)
	
	⊕ ⊕ OO	⊕ ⊕ ⊕ O	⊕ ⊕ ⊕ ⊕

Diuretics vs. alpha-blockers	0.89 (0.31 to 2.52)	0.51 (0.40 to 0.64)	-
	
	⊕ OOO	⊕ ⊕ ⊕ O	

Diuretics vs. ARB	0.86 (0.39 to 3.27)	0.80 (0.61 to 0.98)	1.59 (1.23 to 2.12)
	
	⊕ OOO	⊕ ⊕ OO	⊕ ⊕ OO

BB vs. ACE	1.03 (0.17 to 3.76)	1.21 (0.91 to 1.69)	1.31 (0.95 to 1.88)
	
	⊕ OOO	⊕ OOO	⊕ OOO

BB vs. CCB	1.10 (0.23 to 3.31)	1.00 (0.76 to 1.33)	1.17 (0.89 to 1.61)
	
	⊕ OOO	⊕ OOO	⊕ OOO

BB vs. alpha-blockers	0.93 (0.11 to 4.35)	0.69 (0.50 to 1.02)	-
	
	⊕ OOO	⊕ OOO	

BB vs. ARB	0.88 (0.31 to 2.58)	1.08 (0.86 to 1.38)	1.46 (1.15 to 1.98)
	
	⊕ ⊕ OO	⊕ ⊕ ⊕ ⊕	⊕ ⊕ ⊕ ⊕

ACE vs. CCB	1.08 (0.48 to 2.44)	0.82 (0.69 to 0.94)	0.89 (0.73 to 1.10)
	
	⊕ OOO	⊕ ⊕ ⊕ O	⊕ ⊕ OO

ACE vs. alpha-blockers	0.91 (0.22 to 3.42)	0.58 (0.43 to 0.75)	-
	
	⊕ OOO	⊕ ⊕ OO	

ACE vs. ARB	0.86 (0.35 to 3.50)	0.90 (0.67 to 1.10)	1.11 (0.85 to 1.51)
	
	⊕ OOO	⊕ OOO	⊕ OOO

CCB vs. alpha-blockers	0.85 (0.23 to 2.78)	0.70 (0.53 to 0.92)	-
	
	⊕ OOO	⊕ ⊕ OO	

CCB vs. ARB	0.81 (0.45 to 2.30)	1.10 (0.87 to 1.31)	1.25 (1.02 to 1.56)
	
	⊕ ⊕ OO	⊕ ⊕ ⊕ O	⊕ ⊕ ⊕ ⊕

Alpha-blockers vs. ARB	0.95 (0.29 to 5.71)	1.57 (1.09 to 2.12)	-
	
	⊕ OOO	⊕ ⊕ OO	

ACE, Angiotensin Converting Enzyme Inhibitors; ARB, Angiotensin Receptor Blockers; BB, Beta-blockers; CCB, Calcium Channel Blockers; ⊕ ⊕ ⊕ ⊕, High quality evidence; ⊕ ⊕ ⊕ O, Moderate quality evidence; ⊕ ⊕ OO, Low quality evidence; ⊕ OOO, Very low quality evidence

As expected, the results were favoring active drug treatment over placebo or no treatment.

For most drug-drug comparisons, we found few significant differences, and for most comparisons the quality of the evidence was rated as low (or very low). Overall, the results were ambiguous, with no drug-class standing out as superior across different outcomes.

There was high quality evidence that beta-blockers are inferior to ARB in terms of total mortality (RR 1.14; 95% CrI 1.02 to 1.28). Other significant mortality differences represented only low or very low quality evidence.

The inferiority of ACE-inhibitors to CCB regarding stroke-risk was significant and based on moderate quality evidence (RR 1.19; 95% CrI 1.03 to 1.38). Similarly, the superiority of ACE-inhibitors over CCB with regards to risk of developing heart failure was also significant and based on moderate quality evidence (RR 0.82; 95% CrI 0.69 to 0.94).

We found moderate quality evidence that diuretics reduce the risk of myocardial infarction, compared to beta-blockers (RR 0.82; 95% CrI 0.68 to 0.98). Diuretics were also, based on moderate quality evidence, significantly better at reducing the risk of heart failure than CCB (RR 0.73; 95% CrI 0.62 to 0.84), beta-blockers (RR 0.73; 95% CrI 0.54 to 0.96) and alpha-blockers (RR 0.51; 95% CrI 0.40 to 0.64). Diuretics, however, significantly increased the risk of diabetes relative to ACE-inhibitors (RR 1.43; 95% CrI 1.12 to 1.83) and CCB (RR 1.27; 95% CrI 1.05 to 1.57), based on moderate and high quality evidence, respectively.

#### Ranking of drug-classes

Consistent with our effect estimates per comparison (Tables 3 and 4), the findings from our ranking of drug-classes are ambiguous in the sense that certain drug-classes were superior for some outcomes, while other drugs fared better for other outcomes (Table 5).

**Table 5:** Proportion of times that a drug-class ended up among the top three (in repeated simulations)

	Mortality	Myocardial infarction	Stroke	Angina	Heart failure	Diabetes
Diuretics	18%	79%	46%	33%	99%	1%

BB	1%	2%	3%	38%	10%	9%

ACE	23%	75%	11%	27%	83%	96%

CCB	54%	36%	98%	51%	1%	62%

Alpha-blocker	25%	58%	9%	45%	0%	-

ARB	49%	5%	60%	22%	22%	99%

Diuretics and/or BB	84%	45%	71%	22%	65%	33%

"Conventional"	46%	0%	1%	-	20%	-

ACE, Angiotensin Converting Enzyme Inhibitors; ARB, Angiotensin Receptor Blockers; BB, Beta-blockers; CCB, Calcium Channel Blockers;

#### Consistency of network-model

We did not find any statistically significant inconsistencies in the network when comparing effect estimates based on direct vs. indirect evidence. However, there were some inconsistencies that we should point out (Table 6):

**Table 6:** Effect estimates from multiple-treatment meta-analysis (MTM) compared to direct and indirect estimates, based on node-splitting [23]

	Outcome	MTM-estimate (95% Crl)	Direct estimate (95% Crl)	Indirect estimate (95% Crl)	*P*-value for inconsistency
Diuretics vs. beta-blockers	Diabetes- incidence	1.09	0.88	1.31	0.13
		(0.80 to 1.44)	(0.59 to 1.32)	(0.88 to 2.17)	

Diuretics vs. ACE-inhibitors	Diabetes- incidence	1.43	1.54	1.25	0.24
		(1.12 to 1.83)	(1.14 to 2.10)	(0.89 to 1.79)	

Diuretics vs. placebo	All-cause mortality	0.88	0.89	0.80	0.29
		(0.80 to 0.95)	(0.82 to 0.97)	(0.68 to 0.96)	

Beta-blockers vs. ARB	Stroke	1.23	1.34	1.00	0.17
		(0.96 to 1.49)	(1.03 to 1.74)	(0.69 to 1.41)	

Beta-blockers vs. ARB	Diabetes- incidence	1.46	1.32	1.98	0.13
		(1.15 to 1.98)	(0.97 to 1.82)	(1.26 to 3.40)	

ACE- inhibitors vs. CCB	All-cause mortality	1.02	1.05	0.95	0.21
		(0.95 to 1.10)	(0.96 to 1.13)	(0.82 to 1.09)	

ACE- inhibitors vs. CCB	Heart fail ure	0.82	0.79	0.68	0.29
		(0.69 to 0.94)	(0.67 to 0.95)	(0.52 to 0.90)	

ACE-inhibitors vs. placebo	All-cause mortality	0.87	1.30	0.86	0.08
		(0.79 to 0.96)	(0.82 to 2.14)	(0.77 to 0.95)	

Diuretics and/or beta-blockers vs. placebo	All-cause mortality	0.82	0.57	0.85	0.06
		(0.73 to 0.92)	(0.34 to 0.84)	(0.75 to 0.96)	

CCB vs. ARB	Stroke	0.91	0.86	1.15	0.19
		(0.75 to 1.11)	(0.69 to 1.05)	(0.78 to 1.70)	

CCB vs. ARB	Diabetes- incidence	1.25	1.29	0.86	0.14
		(1.02 to 1.56)	(1.07 to 1.71)	(0.49 to 1.49)	

Table is limited to comparisons where Bayesian "*P*-values" for inconsistency were < 0.3. For a complete overview of MTM-, direct, and indirect estimates see Additional file 6

In three instances the inclusion of indirect evidence shifted the effect estimate from "non-significant" (that is, the CrI included the value 1) to "significant", or vice versa. First, for beta-blockers vs. ARB the direct comparison-analysis yielded a significant increased risk of stroke with beta-blockers (RR 1.34, Crl 1.03 to 1.74), whereas the result from the MTM did not (RR 1.23, Crl 0.96 to 1.49) (Bayesian *P*-value for inconsistency = 0.17). Second, also for beta-blockers vs. ARB, the results for diabetes incidence based on direct evidence was not significant (RR.32, Crl 0.97 to 1.82), while in the MTM it was (RR 1.46, Crl 1.15 to 1.98) (Bayesian *P*-value for inconsistency = 0.13). Third, for the comparison of ACE-inhibitors vs. placebo the direct evidence-analysis yielded an insignificant difference for all-cause mortality (RR 1.30, Crl 0.82 to 2.14), which became significant in favor of ACE-inhibitors in the MTM (RR posterior median 0.87, Crl 0.79 to 0.96) (Bayesian *P*-value for inconsistency = 0.08).

The lowest *P*-value (0.06) for inconsistency was seen for all-cause mortality in the diuretics and/or beta-blockers vs. placebo-comparison. In this case, both estimates favored diuretics and/or beta-blockers significantly, but the effect size estimates differed (direct evidence: RR 0.57, Crl 0.34 to 0.84; MTM: RR 0.82, Crl 0.73 to 0.93).

The absence of clear inconsistencies in the network suggests that our model is trustworthy, but some caution is warranted when interpreting the findings that changed substantially after the inclusion of indirect evidence. The full table of comparisons between results from MTM and results based on direct and indirect evidence are shown in Additional file 6.

## Discussion

### Summary of evidence

Our analysis is, to date, the most comprehensive analysis of the existing data on the comparative effectiveness of different antihypertensive drug-classes used in primary prevention of cardiovascular diseases.

As for most other systematic reviews in this field we find limited evidence of important differences between the various drug-classes. The differences we do find are not easy to put into practice as the ranking of a drug-class depends on which outcome one chooses to emphasize, and no drugs are consistently among the best across all important outcomes.

Our ranking of drug-classes, as presented in Table 5 may be useful to decision makers, or it may add to the confusion. By presenting the chance that a drug is among the top three for an outcome, we had hoped that one or two drugs would emerge as first choice candidates by being among the three best drugs across several important outcomes. However, no such pattern appeared. We should also point out that the quality of the underlying evidence is not taken into consideration in the ranking, thus the results should be interpreted cautiously, and in conjunction with the drug-comparison findings (Tables 3 and 4).

Beta-blockers (atenolol) were inferior to all drug-classes for all primary outcomes, and although the difference in many cases was non-significant and the quality of the evidence was mixed, this may be seen as evidence against opting for these drugs as the first choice. Beta-blockers and alpha-blockers were the only drug-classes that were not significantly superior to any drug, for any outcome, which could suggest not recommending these as first line medication.

### Clinical inferences

Successful management of hypertension is dependent on many factors, and choice of drug class is one that seems to be of limited importance. Thus, clinicians should probably focus more on issues such as limiting adverse events, improving adherence and better follow up of patients rather than on which drug to select. However, there is considerable variation in costs across different antihypertensive agents, thus cost-effectiveness assessments may be important for decisions about choice of medications.

### Our findings in relation to other systematic reviews

Other research groups have conducted network meta-analyses in this field before us, but our contribution adds important dimensions. First, some reviewers have only included one clinical outcome, while we included six clinically important ones. Second, others have reported only on selected drug-drug comparisons [8,56], rather than the full range of competing options. Third, a weakness across earlier reviews of the comparative effectiveness of different antihypertensive drugs is that they have not included an explicit assessment of the quality of the evidence backing the reported effect estimates. An important exception is the systematic review that informed the recently updated guidance from the National Institute of Health and Clinical Excellence (NICE), but their effect-estimates were based on the traditional, not the network meta-analytical approach [9,57].

Although disagreements between our findings and those of other systematic reviews are few and relatively minor, the conclusions drawn by authors vary somewhat [5,6,8,9,12-14,56,58,59].

In two recent network analyses on the effectiveness of antihypertensive drugs, the authors limited their analysis to one outcome: heart failure [14] and diabetes incidence [13]. Despite slightly different study inclusion criteria, their effect estimates are very similar to ours. The systematic review and network meta-analysis by Psaty and colleagues only included comparisons against diuretics, not between other types of antihypertensive drugs [8]. A network analysis by Aursnes and colleagues, also from 2003, focussed on comparing ACE-inhibitors and CCB, and was limited to three outcomes [56]. Our findings are not in full agreement with these two earlier reports, presumably due to our more strict inclusion criteria and perhaps also to the inclusion of results from more recent studies.

Law and colleagues authored a recent comprehensive review and meta-analysis on antihypertensive drug treatment [6]. They conducted traditional meta-analyses, without the network approach. Their conclusion was that "all the classes of blood pressure lowering drugs have a similar effect in reducing CHD (coronary heart disease) effects and stroke". This is close to, but not entirely in agreement with our findings, which may be due to some of the following issues. First, they elected to compare each drug class with the pooled results from all other drug classes, for example, beta-blockers versus all non-beta-blockers. This analytical approach can be misleading because favorable effects from one non-beta-blocker may be off-set by unfavorable effects from another non-beta-blocker drug-class. Second, they included trials where high dose diuretics were used. This may be misleading as there are good reasons to believe that high dose diuretics lead to less favorable outcomes than low dose diuretics [7]. Consequently, as high dose diuretics were used in many of the trials comparing beta-blockers and diuretics, beta-blockers came out more favorably in their analyses than they probably should. Third, in two studies included in their analysis the participants were randomised to either active drug or placebo [54,55], and these should, therefore, not be classified as drug comparison studies, in our view. Fourth, they did not explicitly assess the quality of the evidence underlying their effect-estimates, which is essential for judgements about how confident we can be about the validity of the findings. Last, they included two studies we classified as non-randomised trials [60,61].

### Limitations

Systematic reviews, as other types of research, are inevitably based on subjective judgements. The assessments were, however, done by at least two reviewers, making misjudgements less likely, but still possible.

Although the process of grading the quality of the evidence was done using a structured approach (GRADE), the assessments are strongly influenced by our judgements. The merit of the GRADE-system is that these judgements are made explicit and accounted for.

As with other research activities, systematic reviews do not provide answers to questions not asked by the authors. We have selected the interventions and outcomes we considered most important, but there are undoubtedly other aspects that are important for decision-making in this field. We have, for example, not reviewed the side-effect profiles of the different drug-classes (except for incidence of diabetes). Our selection of outcomes is also debatable. We chose to emphasize what we considered the most important clinical outcomes and disregarded others that may be of key interest, such as intermittent claudication, vascular dementia, renal disease and retinal disease.

A fundamental challenge with the use of meta-analysis is to judge whether two or more studies are sufficiently similar to have their results pooled in one analysis. Our judgements regarding this could be criticized. It is, for instance, not obvious that drugs from the same drug-class are equivalent, as we have implicitly assumed [11,62]. We had specifically planned to conduct a separate analysis where we excluded trials of beta-blockers that had used the agent atenolol, since the appropriateness of using atenolol as a comparator-drug has been questioned [11]. However, atenolol was used in all the beta-blocker trials we included in our review, so an analysis of non-atenolol beta-blockers could not be done. Similarly, our handling of calcium channel blockers as an entity can be questioned. The pharmacological properties vary across these drugs, and it could be argued that they should be grouped according to property and not as an entity [63].

In a network analysis like ours, it is assumed that all the included trials are sufficiently homogeneous to allow for the combining of all the study findings into one analysis. This assumption is difficult to validate. Differences in study populations can, in particular, distort estimates for the effects on total mortality, since these are related to the proportion of deaths that are due to cardiovascular diseases, for each study. We did not formally assess how comparable the various populations were. However, our use of relatively strict inclusion criteria, for example, including only studies where the majority of participants had no prior cardiovascular event and excluding studies of specific high-risk groups, substantiates that the populations were somewhat similar. Also, the finding that the effect-estimates from the network analysis were similar to the estimates from the direct comparisons provides some evidence that the trials were reasonably homogeneous.

The definitions of outcomes vary from study to study, for instance, regarding heart failure. Study reports are not always clear with respect to whether the number of patients with events or the total number of events were counted. We believe such differences across studies has had limited influence on our overall findings.

Our objective was to estimate the relative risk reduction for different antihypertensive drugs in individuals without cardiovascular disease (primary prevention). However, we chose to include studies where up to half the participants had experienced a cardiovascular event (secondary prevention). Our reasoning was that such studies contain information of relevance to our research question. This is in accordance with the approach used by the World Health Organization when they prepared their most recent guidelines on primary prevention of cardiovascular diseases [64]. In several earlier reviews on antihypertensive treatment the authors have included studies where all participants were patients with cardiovascular disease. This is clearly valid if the relative effect of using antihypertensive medication is the same for healthy and for sick people, and Law and colleagues do provide some data to support such a view [6]. Our choice to exclude such trials may thus be criticized. On the other hand, it is not firmly established that the relative treatment effect of antihypertensive medication is constant across different patient groups. And even if this proves to be the case it is still conceivable that different types of medication may work differently for people with and without cardiovascular disease, something for which Law and colleagues also found evidence [6].

Our meta-analyses are based on a count of events at one moment in time, that is, at the end of each trial. This analytic approach is not entirely valid unless the relative effect size is constant over time. A more complex analysis, including time to events, would require access to more data from each study than what was available to us.

The majority of the included trials in this review were sponsored by companies with a vested interest in the study results. Such sponsorship has been associated with bias in favour of the product made by the funding company [65]. Possible explanations include publication bias and use of inappropriate comparators. Limiting our review to large-scale studies should reduce risk of publication bias or other forms of selective reporting [20]. Whether the most appropriate comparator drug has been selected is more difficult to assess. Biased analyses were minimized in our review because we based our effect-estimates on actual figures presented in the various articles, rather than relying on the analyses conducted by the study-authors and/or sponsors.

### Future research agenda

Despite the fact that many methodologically sound large-scale trials of anti-hypertensive drugs have been conducted, our confidence in the overall findings ranged from very low to high after assessing the quality of the evidence using the GRADE-instrument. This means that the results from future trials may alter our conclusions. Future research to improve the quality of hypertension management should also focus on other issues, such as interventions to improve treatment adherence and on how to organise follow-up of patients more effectively.

## Conclusions

Based on the available evidence, there seems to be little or no difference between commonly used blood pressure lowering medications with regards cardiovascular risk reduction. Beta-blockers (atenolol) and alpha-blockers are the only drug-classes that were not significantly superior to any other drugs, for any outcome, and may thus not be prime candidates for first-line antihypertensive treatment.

## Abbreviations

ACE: angiotensin convering enzyme; ARB: angiotensin receptor blockers; CCB: calcium-channel blockers; CrI: credibility interval; GRADE: Grading of Recommendations Assessment: Development and Evaluation; MTM: Multiple Treatments Meta-analysis; RR: relative risk.

## Competing interests

AF, JOJ, OB, IN, OFN, AS, HT and SF declare they have no competing interests. SM is an employee of the Norwegian Medicines Agency that has a strong interest in the cost-effective use of medication. ISK has received salary, consultancy honoraria, gifts and travel funding from a wide range of public institutions, private companies and patient organizations that may have an interest in antihypertensive treatment.

## Authors' contributions

All authors contributed to the planning of this review, and all, except JOJ, participated in the trial-selection process. JOJ and AF conducted the analyses, and AF wrote the first draft of the article. All authors read and provided feed-back on the draft-versions of the article, and approved the final version. AF is the guarantor.

## Pre-publication history

The pre-publication history for this paper can be accessed here:

http://www.biomedcentral.com/1741-7015/10/33/prepub

## Supplementary Material

Additional file 1 **Strategies for electronic searches in databases**. This file contains detailed descriptions of the search-strategies used in the various electronic databases that were searched to identify relevant randomized controlled trials. Click here for file

Additional file 2 **Table of excluded studies**. Contains a list of studies that were excluded from the review, mostly after reading the full text reports (articles excluded after reading title/abstract are generally not included). The causes for exclusion are also listed. Click here for file

Additional file 3 **Main findings (primary outcomes)**. Full, comprehensive version of Table 3. Click here for file

Additional file 4 **Main findings (secondary outcomes)**. Full, comprehensive version of Table 4. Click here for file

Additional file 5 **GRADE-profiles**. In this file, the reasoning for our grading of the evidence is presented, for all outcomes across all comparisons. Click here for file

Additional file 6 **Estimates of effect from multiple-treatment meta-analysis (MTM) compared to the direct and indirect estimates of effect based on node-splitting**. Full, comprehensive version of Table 6. Click here for file
