# Supplementary material for: Comparative effectiveness of antihypertensive medication for primary prevention of cardiovascular disease: systematic review and multiple treatments meta-analysis
Source: BMC Med. 2012 Apr 5;10:33. doi: 10.1186/1741-7015-10-33 (PMC3354999; doi:10.1186/1741-7015-10-33)
Supplement: Additional file 1 — Strategies for electronic searches in databases. This file contains detailed descriptions of the search-strategies used in the various electronic databases that were searched to identify relevant randomized controlled trials. [file 1741-7015-10-33-S1.DOC]

# Additional file 1 (Fretheim et al 2012)

# Strategies for electronic searches

Contents

[1. CENTRAL 2](#__RefHeading___Toc248761899)

[2. MEDLINE (Preventive and pharmaceutical treatment) 5](#__RefHeading___Toc248761900)

[3. MEDLINE (Alternative treatment) 6](#__RefHeading___Toc248761901)

[4. EMBASE (Preventive and pharmaceutical treatment) 8](#__RefHeading___Toc248761902)

[5. EMBASE (Alternative treatment) 9](#__RefHeading___Toc248761903)

[6. AMED (Preventive and pharmaceutical treatment) 11](#__RefHeading___Toc248761904)

# **1. CENTRAL**

#1 MeSH descriptor Hypertension explode all trees with qualifiers: DT,DH,PC

#2 MeSH descriptor Diabetes Mellitus, Type 2 explode all trees with qualifiers: DT,DH,PC

#3 MeSH descriptor Smoking explode all trees with qualifiers: DT,DH,PC

#4 MeSH descriptor Albuminuria, this term only with qualifiers: DT,DH,PC

#5 MeSH descriptor Cardiomegaly explode all trees with qualifiers: DT,DH,PC

#6 MeSH descriptor Hyperlipidemias explode all trees with qualifiers: DT,DH,PC

#7 MeSH descriptor Hypertension explode all trees

#8 MeSH descriptor Diabetes Mellitus, Type 2 explode all trees

#9 MeSH descriptor Smoking explode all trees

#10 MeSH descriptor Albuminuria, this term only

#11 MeSH descriptor Cardiomegaly explode all trees

#12 MeSH descriptor Hyperlipidemias explode all trees

#13 (hyperlipidemia? or hypertension or "high blood pressure" or "diabetes mellitus" or diabetic? or albuminura? or cardiomegaly or enlarge NEAR/2 heart? or smoking or smoker?):ti,ab

#14 MeSH descriptor Antihypertensive Agents explode all trees

#15 MeSH descriptor Adrenergic beta-Antagonists explode all trees

#16 MeSH descriptor Angiotensin-Converting Enzyme Inhibitors explode all trees

#17 MeSH descriptor Calcium Channel Blockers explode all trees

#18 MeSH descriptor Diuretics explode all trees

#19 MeSH descriptor Anticholesteremic Agents explode all trees

#20 MeSH descriptor Hydroxymethylglutaryl-CoA Reductase Inhibitors explode all trees

#21 MeSH descriptor Antilipemic Agents explode all trees

#22 MeSH descriptor Hypoglycemic Agents explode all trees

#23 MeSH descriptor Thrombolytic Therapy explode all trees

#24 ("anti hypertensive" or antihypertencive or beta NEAR antagonist? or beta NEAR block* or angiotensin NEAR/2 antagonist? or angiotensin NEAR/2 inhibitor? or ace NEAR/2 antagonist? or ace NEAR/2 inhibitor? or kininase NEAR/2 antagonist? or kininase NEAR/2 inhibitor? or calcium NEAR/2 blocker? or calcium NEAR/2 inhibitor? or calcium NEAR/2 antagonist? or diuretics or anticholesteremic or hypocholesteremic or cholesterol NEXT inhibitor? or cholesterol NEAR/2 lowering or cholesterol NEAR reduc* or lipid NEAR/2 lowering or lipid NEAR/2 reduc* or statin? or hydroxymethylglutaryl NEAR/2 inhibitor? or hmg NEAR/2 inhibitor? or antilipemic? or anti NEXT lipemic? or antihyperlipidemic? or anti NEXT hyperlipidemic? or hypolipidemic? or hypoglycemic? or anti NEXT diabetic? or antidiabetic? or thromboly* NEXT therap* or fibrinolytic NEXT therap*):ti,ab

#25 MeSH descriptor Diet explode all trees

#26 MeSH descriptor Diet Therapy explode all trees

#27 MeSH descriptor Dietary Fats explode all trees

#28 MeSH descriptor Dietary Supplements explode all trees

#29 MeSH descriptor Fish Oils explode all trees

#30 MeSH descriptor Antioxidants explode all trees

#31 MeSH descriptor Tobacco Use Cessation explode all trees

#32 MeSH descriptor Exercise explode all trees

#33 MeSH descriptor Exercise Therapy explode all trees

#34 MeSH descriptor Sports explode all trees

#35 MeSH descriptor Weight Loss, this term only

#36 MeSH descriptor Complementary Therapies explode all trees

#37 (diet? or dietary NEXT fat? or diatary NEAR/2 supplement? or food NEAR/2 supplement? or nutraceutical? or neutraceutical? or fish NEAR/2 oil? or cod NEAR/2 oil? or "omega 3" or antioxidant? or tobacco NEAR/2 cessation? or smoke* NEAR/2 cessation? or smoke* NEAR/2 stop* or smoke* NEAR/2 reduc* or exrercise? or sport? or weight NEAR/2 loss* or weight NEAR/2 lose or weight NEAR/2 reduc* or "complementary medicine" or complementary NEXT therap* or "alternative medicine"):ti,ab

#38 MeSH descriptor Cardiovascular Diseases, this term only

#39 MeSH descriptor Heart Failure, this term only

#40 MeSH descriptor Myocardial Ischemia, this term only

#41 MeSH descriptor Acute Coronary Syndrome, this term only

#42 MeSH descriptor Angina, Unstable, this term only

#43 MeSH descriptor Coronary Disease, this term only

#44 MeSH descriptor Coronary Aneurysm, this term only

#45 MeSH descriptor Coronary Artery Disease, this term only

#46 MeSH descriptor Coronary Occlusion, this term only

#47 MeSH descriptor Coronary Stenosis, this term only

#48 MeSH descriptor Coronary Restenosis, this term only

#49 MeSH descriptor Coronary Thrombosis, this term only

#50 MeSH descriptor Myocardial Infarction, this term only

#51 MeSH descriptor Cerebrovascular Disorders, this term only

#52 MeSH descriptor Brain Ischemia, this term only

#53 MeSH descriptor Brain Infarction, this term only

#54 MeSH descriptor Cerebral Infarction, this term only

#55 MeSH descriptor Ischemic Attack, Transient, this term only

#56 MeSH descriptor Infarction, Anterior Cerebral Artery, this term only

#57 MeSH descriptor Infarction, Middle Cerebral Artery, this term only

#58 MeSH descriptor Infarction, Posterior Cerebral Artery, this term only

#59 MeSH descriptor Intracranial Embolism and Thrombosis, this term only

#60 MeSH descriptor Intracranial Embolism, this term only

#61 MeSH descriptor Intracranial Thrombosis, this term only

#62 MeSH descriptor Intracranial Hemorrhages, this term only

#63 MeSH descriptor Cerebral Hemorrhage, this term only

#64 MeSH descriptor Intracranial Hemorrhage, Hypertensive, this term only

#65 MeSH descriptor Stroke, this term only

#66 MeSH descriptor Angina Pectoris, this term only

#67 (cerebrovascular NEXT disorder? or cerebrovascular NEXT disease? or vascular NEXT disorder? or vascular NEXT disease? or cardiovascular NEXT disorder? or cardiovascular NEXT disease? or heart NEXT disease* or heart NEXT disorder? or heart NEXT failure? or cardiac NEXT disease* or cardiac NEXT disorder? or coronary NEAR/2 disease? or coronary NEAR/2 disorder? or coronary NEAR/2 syndrome or coronary NEAR/2 aneurysm or coronary NEAR/2 occlusion or coronary NEAR/2 stenosis or coronary NEAR/2 restenosos or cerebral NEXT hemorrhage or intracranial NEXT hemorrhage or angina or stroke? or apoplexy or infarct* or ischemia or ischemic or thrombos* or embolism):ti,ab

#68 MeSH descriptor Cardiovascular Diseases, this term only with qualifiers: DT,DH,PC

#69 MeSH descriptor Heart Failure, this term only with qualifiers: DT,DH,PC

#70 MeSH descriptor Myocardial Ischemia, this term only with qualifiers: DT,DH,PC

#71 MeSH descriptor Acute Coronary Syndrome, this term only with qualifiers: DT,DH,PC

#72 MeSH descriptor Angina, Unstable, this term only with qualifiers: DT,DH,PC

#73 MeSH descriptor Coronary Disease, this term only with qualifiers: DT,DH,PC

#74 MeSH descriptor Coronary Aneurysm, this term only with qualifiers: DT,DH,PC

#75 MeSH descriptor Coronary Artery Disease, this term only with qualifiers: DT,DH,PC

#76 MeSH descriptor Coronary Occlusion, this term only with qualifiers: DT,DH,PC

#77 MeSH descriptor Coronary Stenosis, this term only with qualifiers: DT,DH,PC

#78 MeSH descriptor Coronary Restenosis, this term only with qualifiers: DT,DH,PC

#79 MeSH descriptor Coronary Thrombosis, this term only with qualifiers: DT,DH,PC

#80 MeSH descriptor Myocardial Infarction, this term only with qualifiers: DT,DH,PC

#81 MeSH descriptor Cerebrovascular Disorders, this term only with qualifiers: DT,DH,PC

#82 MeSH descriptor Brain Ischemia, this term only with qualifiers: DT,DH,PC

#83 MeSH descriptor Brain Infarction, this term only with qualifiers: DT,DH,PC

#84 MeSH descriptor Cerebral Infarction, this term only with qualifiers: DT,DH,PC

#85 MeSH descriptor Ischemic Attack, Transient, this term only with qualifiers: DT,DH,PC

#86 MeSH descriptor Infarction, Anterior Cerebral Artery, this term only with qualifiers: DT,DH,PC

#87 MeSH descriptor Infarction, Middle Cerebral Artery, this term only with qualifiers: DT,DH,PC

#88 MeSH descriptor Infarction, Posterior Cerebral Artery, this term only with qualifiers: DT,DH,PC

#89 MeSH descriptor Intracranial Embolism and Thrombosis, this term only with qualifiers: DT,DH,PC

#90 MeSH descriptor Intracranial Embolism, this term only with qualifiers: DT,DH,PC

#91 MeSH descriptor Intracranial Thrombosis, this term only with qualifiers: DT,DH,PC

#92 MeSH descriptor Intracranial Hemorrhages, this term only with qualifiers: DT,DH,PC

#93 MeSH descriptor Cerebral Hemorrhage, this term only with qualifiers: DT,DH,PC

#94 MeSH descriptor Intracranial Hemorrhage, Hypertensive, this term only with qualifiers: DT,DH,PC

#95 MeSH descriptor Stroke, this term only with qualifiers: DT,DH,PC

#96 MeSH descriptor Angina Pectoris, this term only with qualifiers: DT,DH,PC

#97 (#1 OR #2 OR #3 OR #4 OR #5 OR #6)

#98 (#7 OR #8 OR #9 OR #10 OR #11 OR #12 OR #13)

#99 (#14 OR #15 OR #16 OR #17 OR #18 OR #19 OR #20 OR #21 OR #22 OR #23 OR #24 OR #25 OR #26 OR #27 OR #28 OR #29 OR #30 OR #31 OR #32 OR #33 OR #34 OR #35 OR #36 OR #37)

#100 (#38 OR #39 OR #40 OR #41 OR #42 OR #43 OR #44 OR #45 OR #46 OR #47 OR #48 OR #49 OR #50 OR #51 OR #52 OR #53 OR #54 OR #55 OR #56 OR #57 OR #58 OR #59 OR #60 OR #61 OR #62 OR #63 OR #64 OR #65 OR #66 OR #67)

#101 (#68 OR #69 OR #70 OR #71 OR #72 OR #73 OR #74 OR #75 OR #76 OR #77 OR #78 OR #79 OR #80 OR #81 OR #82 OR #83 OR #84 OR #85 OR #86 OR #87 OR #88 OR #89 OR #90 OR #91 OR #92 OR #93 OR #94 OR #95 OR #96)

#102 (#98 AND #99 AND #100)

#103 (#97 AND #100)

#104 (#98 AND #101)

#105 (#102 OR #103 OR #104)

# 2. MEDLINE (Preventive and pharmaceutical treatment)

1. exp hyperlipidemia/dt or exp hypertension/dt or exp diabetes mellitus, type 2/dt or Albuminuria/dt or exp Cardiomegaly/dt

2. Antihypertensive Agents/

3. Adrenergic beta-Antagonists/

4. Angiotensin-Converting Enzyme Inhibitors/

5. Calcium Channel Blockers/

6. exp Diuretics/

7. Anticholesteremic Agents/

8. Hydroxymethylglutaryl-CoA Reductase Inhibitors/

9. Antilipemic Agents/

10. statin$1.tw.

11. ((lipid-lowering or (lipid adj lowering) or cholesterol-lowering or (cholesterol adj lowering)) and (agent$1 or drug$1)).tw.

12. Hypoglycemic Agents/

13. ((Antidiabetic or Hypoglycemic$1) and (drug$1 or agent$1)).tw.

14. Platelet Aggregation Inhibitors/

15. aspirin/

16. or/2-15

17. 16 and tu.fs.

18. 1 and 17

19. Cardiovascular Diseases/

20. Heart Diseases/ or Heart Arrest/

21. Heart Failure, Congestive/ or exp Angina Pectoris/

22. Coronary Disease/

23. Myocardial Ischemia/ or Myocardial Infarction/ or Shock, Cardiogenic/ or exp Myocardial Revascularization/

24. Cerebrovascular Disorders/

25. Cerebrovascular Accident/

26. Cerebral Hemorrhage/ or Brain Ischemia/ or Intracranial Hemorrhage/ or Cerebral Hemorrhage/

27. exp Brain Infarction/ or exp Cerebral Arterial Disease/ or exp "Intracranial Embolism and Thrombosis"/ or exp Cerebral Infarction/

28. Death/ or Death, Sudden/

29. or/19-28

30. or/19-28

31. 18 and 30

32. randomized controlled trial.pt.

33. Randomized Controlled Trials/

34. Random Allocation/

35. Double-Blind Method/

36. Single-Blind Method/

37. clinical trial.pt.

38. exp clinical trials/ or intervention studies/ or (intervention$ adj (stud$4 or trial$1)).tw.

39. (clinic$ adj trial$1).tw.

40. ((singl$ or doubl$ or treb$ or tripl$) adj (blind$3 or mask$3)).tw.

41. PLACEBOS/

42. placebo$.tw.

43. randomly allocated.tw.

44. random$.tw.

45. or/32-44

46. case report.tw.

47. (letter or editorial).pt.

48. historical article.pt.

49. review of reported cases.pt.

50. review, multicase.pt. or review.sh. or review.pt.

51. animal/

52. human/

53. 51 not (51 and 52)

54. or/46-50,53

55. 45 not 54

56. Meta-analysis/

57. meta analy$.tw.

58. metaanaly$.tw.

59. meta analysis.pt.

60. (systematic adj (review$1 or overview$1)).tw.

61. cochrane.tw. or 1469-493x.is.

62. or/56-61

63. 31 and 55

64. 31 and 62

65. 63 or 64

# 3. MEDLINE (Alternative treatment)

1. Exercise/ or Diet/ or Diet, Reducing/ or Weight Loss/ or Smoking cessation/

2. (physical adj activity).tw.

3. Complementary Therapies/

4. ((complementary or alternative) and (therap$3 or medicine)).tw.

5. or/1-4

6. Cardiovascular Diseases/

7. Heart Diseases/ or Heart Arrest/

8. Heart Failure, Congestive/ or exp Angina Pectoris/

9. Coronary Disease/

10. Myocardial Ischemia/ or Myocardial Infarction/ or Shock, Cardiogenic/ or exp Myocardial Revascularization/

11. Cerebrovascular Disorders/

12. Cerebrovascular Accident/

13. Cerebral Hemorrhage/ or Brain Ischemia/ or Intracranial Hemorrhage/ or Cerebral Hemorrhage/

14. exp Brain Infarction/ or exp Cerebral Arterial Disease/ or exp "Intracranial Embolism and Thrombosis"/ or exp Cerebral Infarction/

15. Death/ or Death, Sudden/

16. or/6-15

17. pc.fs. or (prevent$ or prophyla$).tw.

18. 16 and 17

19. 5 and 18

20. randomized controlled trial.pt.

21. Randomized Controlled Trials/

22. Random Allocation/

23. Double-Blind Method/

24. Single-Blind Method/

25. clinical trial.pt.

26. exp clinical trials/ or intervention studies/ or (intervention$ adj (stud$4 or trial$1)).tw.

27. (clinic$ adj trial$1).tw.

28. ((singl$ or doubl$ or treb$ or tripl$) adj (blind$3 or mask$3)).tw.

29. PLACEBOS/

30. placebo$.tw.

31. randomly allocated.tw.

32. (allocated adj2 random).tw.

33. or/20-32

34. case report.tw.

35. (letter or editorial).pt.

36. historical article.pt.

37. review of reported cases.pt.

38. review, multicase.pt. or review.sh. or review.pt.

39. animal/

40. human/

41. 39 not (39 and 40)

42. or/34-38,41

43. 33 not 42

44. Meta-analysis/

45. meta analy$.tw.

46. metaanaly$.tw.

47. meta analysis.pt.

48. (systematic adj (review$1 or overview$1)).tw.

49. cochrane.tw. or 1469-493x.is.

50. or/44-49

51. 19 and 43

52. 19 and 50

53. 51 or 52

# 4. EMBASE (Preventive and pharmaceutical treatment)

1. exp Hyperlipidemia/dt or exp Hypertension/dt or exp Hypercholesterolemia/dt or exp Non Insulin Dependent Diabetes Mellitus/dt or Albuminuria/dt or Cardiomegaly/dt

2. Antihypertensive Agent/

3. Adrenergic beta-Antagonists/

4. Angiotensin-Converting Enzyme Inhibitors/

5. Calcium Channel Blockers/

6. exp Diuretics/

7. Anticholesteremic Agents/

8. Hydroxymethylglutaryl Coenzyme a Reductase Inhibitor/

9. Antilipemic Agents/

10. statin$1.tw.

11. ((lipid-lowering or (lipid adj lowering) or cholesterol-lowering or (cholesterol adj lowering)) and (agent$1 or drug$1)).tw.

12. Hypoglycemic Agent/

13. ((Antidiabetic or Hypoglycemic$1) and (drug$1 or agent$1)).tw.

14. Platelet Aggregation Inhibitors/

15. aspirin/

16. or/2-15

17. 16 and dt.fs.

18. 1 and 17

19. Cardiovascular Diseases/

20. Heart Diseases/ or Heart Arrest/

21. Heart Failure, Congestive/ or exp Angina Pectoris/

22. Coronary disease/

23. Myocardial Ischemia/ or Myocardial Infarction/ or Shock, Cardiogenic/ or exp Myocardial Revascularization/

24. Cerebrovascular Disease/

25. Cerebrovascular Accident/

26. Cerebral Hemorrhage/ or Brain Ischemia/ or Intracranial Hemorrhage/ or Intracranial Aneurysm/

27. exp Brain Infarction/ or exp Cerebral Arterial Disease/ or exp "Intracranial Embolism and Thrombosis"/ or exp Cerebral Infarction/

28. Death/ or Death, Sudden/

29. or/19-28

30. 18 and 29

31. Clinical Trial/

32. Randomized Controlled Trial/

33. Randomization/

34. Double Blind Procedure/

35. Single Blind Procedure/

36. Crossover Procedure/

37. Placebo/ or intervention studies/ or (intervention$ adj (stud$4 or trial$1)).tw.

38. placebo$.tw.

39. randomi?ed controlled trial$.tw.

40. rct.tw.

41. random allocation.tw.

42. randomly allocated.tw.

43. allocated randomly.tw.

44. (allocated adj2 random).tw.

45. single blind$.tw.

46. double blind$.tw.

47. ((treble or triple) adj blind$).tw.

48. Prospective study/

49. or/31-48

50. Case study/

51. case report.tw. or review$.mp.

52. Abstract report/

53. Letter/ or Editorial/

54. Human/

55. Nonhuman/

56. ANIMAL/

57. Animal Experiment/

58. 55 or 56 or 57

59. 58 not (58 and 54)

60. or/50-53,59

61. 49 not 60

62. Meta-analysis/

63. meta analy$.tw.

64. metaanaly$.tw.

65. Systematic Review/

66. (systematic adj (review$1 or overview$1)).tw.

67. Cochrane Library/ or cochrane.tw. or 1469-493x.is.

68. or/62-67

69. 30 and 61

70. 30 and 68

71. 69 or 70

72. 71 and risk.mp.

# 5. EMBASE (Alternative treatment)

1. Exercise/ or Diet/ or Diet, Reducing/ or Weight Loss/ or Smoking cessation/ or Weight Reduction/

2. (physical adj activity).tw.

3. Complementary Therapies/

4. ((complementary or alternative) and (therap$3 or medicine)).tw.

5. or/1-4

6. Cardiovascular Diseases/

7. Heart Diseases/ or Heart Arrest/

8. Heart Failure, Congestive/ or exp Angina Pectoris/

9. Coronary Disease/

10. Myocardial Ischemia/ or Myocardial Infarction/ or Shock, Cardiogenic/ or exp Myocardial Revascularization/

11. Cerebrovascular Disorders/

12. Cerebrovascular Accident/

13. Cerebral Hemorrhage/ or Brain Ischemia/ or Intracranial Hemorrhage/ or Cerebral Hemorrhage/

14. exp Brain Infarction/ or exp Cerebral Arterial Disease/ or exp "Intracranial Embolism and Thrombosis"/ or exp Cerebral Infarction/

15. Death/ or Death, Sudden/

16. or/6-15

17. pc.fs.

18. 16 and 17

19. 5 and 18

20. Clinical Trial/

21. Randomized Controlled Trial/

22. Randomization/

23. Double Blind Procedure/

24. Double Blind Procedure/

25. Crossover Procedure/

26. Placebo/ or intervention studies/ or (intervention$ adj (stud$4 or trial$1)).tw.

27. placebo$.tw.

28. randomi?ed controlled trial$.tw.

29. rct.tw.

30. random allocation.tw.

31. randomly allocated.tw.

32. allocated randomly.tw.

33. (allocated adj2 random).tw.

34. single blind$.tw.

35. double blind$.tw.

36. ((treble or triple) adj blind$).tw.

37. ((treble or triple) adj blind$).tw.

38. or/20-37

39. Case study/

40. case report.tw. or review$.mp.

41. Abstract report/

42. Abstract report/

43. Human/

44. Nonhuman/

45. ANIMAL/

46. Animal Experiment/

47. 44 or 45 or 46

48. 47 not (43 and 47)

49. or/39-42,48

50. 38 not 49

51. Meta-analysis/

52. meta analy$.tw.

53. metaanaly$.tw.

54. Systematic Review/

55. (systematic adj (review$1 or overview$1)).tw.

56. Cochrane Library/ or cochrane.tw. or 1469-493x.is.

57. or/51-56

58. 19 and 50

59. 19 and 57

60. 58 or 59

61. 60 and risk$.mp.

# 6. AMED (Preventive and pharmaceutical treatment)

1. exp heart disease/

2. exp cerebrovascular disorders/

3. exp cerebral hemorrhage/ or exp cerebral infarction/ or exp cerebral ischemia/

4. death/ or death, sudden/

5. or/1-4

6. exp Antihypertensive Agents/

7. exp adrenergic beta receptor blockaders/

8. (Enzyme inhibitors/ and angiotensin.tw.) or ACE.tw.

9. exp Calcium Channel Blockers/

10. exp Diuretics/

11. exp dietary fats/

12. statin$1.tw.

13. ((lipid or cholesterol) and (lowering or reduc$)).tw.

14. (lipid-lowering or cholesterol-lowering).tw.

15. (antidiabetic$ or antiglycemic$).tw.

16. exp exercise/ or exp physical fitness/

17. exp physical education/ or exp physical endurance/

18. (physical adj activit$).tw.

19. exp diet/ or exp diet therapy/

20. exp diet reducing/

21. exp weight loss/

22. exp smoking/ or exp smoking cessation/

23. exp Risk/ or risk$3.tw. or Treatment Outcome/

24. exp prevention/ or prevent$ pr prophyla$.tw.

25. or/1-4

26. or/6-22

27. 23 and 24

28. 25 and 26 and 27

29. exp random allocation/

30. randomized controlled trials/

31. (random$ or rct).tw.

32. or/29-31

33. 28 and 31

34. 25 and 26 and 32
