# Supplementary material for: Comparative effectiveness of antihypertensive medication for primary prevention of cardiovascular disease: systematic review and multiple treatments meta-analysis
Source: BMC Med. 2012 Apr 5;10:33. doi: 10.1186/1741-7015-10-33 (PMC3354999; doi:10.1186/1741-7015-10-33)
Supplement: Additional file 2 — Table of excluded studies. Contains a list of studies that were excluded from the review, mostly after reading the full text reports (articles excluded after reading title/abstract are generally not included). The causes for exclusion are also listed. [file 1741-7015-10-33-S2.DOC]

# Additional file 2 (Fretheim et al 2012)

# Table of excluded studies

(A selection of excluded studies, mostly excluded after assessment of full-text reports/articles)

| **Name of study or author (year of publication)** | **Cause(s) for exclusion** |
| --- | --- |
| **Wolff & Lindeman (1966)** | Not primary endpoint: Study the feasibility and value of maintaining patients with essential hypertension on effective long-term hypotensive therapy.  Low internal validity. Small study and large proportion not followed up. |
| **VA 1 (1967)** | Medication used was high-dose diuretic |
| **Barraclough (1973)** | Not primary endpoint: Achieved blood pressure level /on treatment blood pressure level. A combination of drugs was used without dosage given. |
| **USPHA (1977)** | Medication used was high-dose diuretic |
| **Morgan (1978)** | Medication used was high-dose diuretic |
| **VA-NHBLI (1978)** | Medication used was high-dose diuretic |
| **ANBP 1 (1980)** | Medication used was high-dose diuretic |
| **Morisky (1983)** | Intervention aimed at improving adherence to hypertension treatment. |
| **Berglund (1986)** | Small trial where only diabetogenic effects were evaluated. |
| **Gøteborg-study (Wilhelmsen 1986)** | The study was populationbased and not a sample of persons with increased risk of CVD. |
| **WHO factory study (1986)** | Population-based study, not targetting high-risk groups. |
| **MRC 1 (1988)** | Medication used was high-dose diuretic |
| **MAPPHY (1988)** | MAPPHY-study included in HAPPHY-study. |
| **SHEP-Pilot (1989)** | Medication used was high-dose diuretic |
| **STARS (Watts) (1992)** | Secondary prevention; not our primary endpoint. |
| **TOMHS (1993)** | Not our primary endpoints. |
| **SCRIP (1994)** | Secondary prevention study. |
| **GLANT (1995)** | Not randomised controlled trial. |
| **KAPS (1995)** | Not our primary endpoints. |
| **CAIUS (1996)** | Not our primary endpoints. |
| **STONE (1996)** | Patients were not randomised, but allocated alternately to treatment groups. |
| **VHAS (1997)** | Not our primary endpoints. |
| **VA-HIT (1999)** | Secondary prevention. |
| **SCAT (2000)** | Not our primary endpoints. |
| **SYST-CHINA (2000)** | Patients were not randomised, but allocated alternately to treatment groups. |
| **HOPE og Micro HOPE (2001)** | 80% with previous myocardial infarction. |
| **Progress (2001)** | Secondary prevention of stroke. |
| **AASK (2001)** | Not relevant endpoints. |
| **IRMA (2001)** | Our endpoints not reported. |
| **ELSA (2002)** | Regression study. |
| **TRIPOD (2002)** | Study population: women with previous gestational diabetes; not our primary endpoints. |
| **Diabetes Prevention Program (2002)** | Not our primary endpoints. |
| **INVEST (2003)** | Secondary prevention. |
| **JMIC-B (2004)** | Secondary prevention. |
| **PREVEND-IT (2004)** | Not our primary endpoints. Also questionable whether the inclusions criteria (microablumiuria identified by population-based screening) is relevant to us. |
| **XENDOS (2004)** | Not our primary endpoints. |
| **FEVER (2005)** | All participants were started on low-dose diuretic medication, and later randomised to placebo or felodipine. This design does not answer questions of direct relevance to our review. |
| **PIPOD (2006)** | Follow-up study of TRIPOD-trial (which was excluded). |
| **CHARISMA (2006)** | More than three quarters of the participants had established cardiovascular disease. |
| **Diabetes Prevention Study (2006)** | Only results on incidence of Type 2 diabetes. |
| **MARPLE (2006)** | Not randomised controlled trial. |
| **ONTARGET (2008)** | Mostly secondary prevention. |
| **TRANSCEND (2008)** | Mostly secondary prevention. |
| **KYOTO Heart study (2009)** | Evaluation of add-on drugs, ARBs vs. non-ARBs. Not addressing our research question. |
