# Supplementary material for: Comparative effectiveness of antihypertensive medication for primary prevention of cardiovascular disease: systematic review and multiple treatments meta-analysis
Source: BMC Med. 2012 Apr 5;10:33. doi: 10.1186/1741-7015-10-33 (PMC3354999; doi:10.1186/1741-7015-10-33)
Supplement: Additional file 3 — Main findings (primary outcomes). Full, comprehensive version of Table 3. [file 1741-7015-10-33-S3.DOC]

**Additional file 3 (Fretheim et al 2012).**

Main findings (primary outcomes).

|  | **All-cause mortality** | **Myocardial infarction** | **Stroke** |
| --- | --- | --- | --- |
| Diuretics vs BB | 0.90 (0.80 to 1.01) | 0.82 (0.68 to 0.98) | 0.83 (0.68 to 1.07) |
| ⊕⊕⊕O | ⊕⊕OO | ⊕⊕OO |
| Diuretics vs ACE | 1.00 (0.93 to 1.08) | 1.00 (0.88 to 1.15) | 0.94 (0.81 to 1.10) |
| ⊕⊕⊕O | ⊕⊕⊕O | ⊕⊕⊕O |
| Diuretics vs CCB | 1.03 (0.96 to 1.10) | 0.96 (0.84 to 1.07) | 1.12 (0.97 to 1.29) |
| ⊕⊕⊕O | ⊕⊕⊕O | ⊕⊕OO |
| Diuretics vs alpha-blockers | 0.98 (0.87 to 1.12) | 0.99 (0.80 to 1.23) | 0.85 (0.66 to 1.12) |
| ⊕⊕⊕O | ⊕⊕⊕O | ⊕⊕OO |
| Diuretics vs ARB | 1.02 (0.92 to 1.14) | 0.83 (0.69 to 1.03) | 1.02 (0.82 to 1.28) |
| ⊕⊕OO | ⊕OOO | ⊕OOO |
| Diuretics vs diuretics and/or BB | 1.07 (0.97 to 1.17) | 0.97 (0.82 to 1.14) | 1.04 (0.87 to 1.25) |
| ⊕⊕OO | ⊕⊕OO | ⊕OOO |
| Diuretics vs ”conventional” | 0.96 (0.25 to 4.12) | 0.37 (0.15 to 0.77) | 0.61 (0.39 to 0.98) |
| ⊕OOO | ⊕⊕⊕O | ⊕⊕OO |
| Diuretics vs placebo | 0.88 (0.80 to 0.95) | 0.76 (0.65 to 0.89) | 0.61 (0.52 to 0.71) |
| ⊕⊕⊕⊕ | ⊕⊕⊕⊕ | ⊕⊕⊕⊕ |
| BB vs ACE | 1.12 (0.98 to 1.27) | 1.22 (1.00 to 1.52) | 1.13 (0.86 to 1.42) |
| ⊕OOO | ⊕⊕OO | ⊕OOO |
| BB vs CCB | 1.14 (1.01 to 1.28) | 1.17 (0.97 to 1.42) | 1.34 (1.05 to 1.64) |
| ⊕⊕OO | ⊕OOO | ⊕⊕OO |
| BB vs alpha-blockers | 1.09 (0.93 to 1.30) | 1.20 (0.92 to 1.61) | 1.02 (0.71 to 1.42) |
| ⊕OOO | ⊕OOO | ⊕OOO |
| BB vs ARB | 1.14 (1.02 to 1.28) | 1.02 (0.84 to 1.27) | 1.23 (0.96 to 1.49) |
| ⊕⊕⊕⊕ | ⊕⊕⊕O | ⊕⊕⊕O |
| BB vs diuretics and/or BB | 1.19 (1.03 to 1.36) | 1.18 (0.95 to 1.48) | 1.24 (0.95 to 1.58) |
| ⊕⊕OO | ⊕OOO | ⊕OOO |
| BB vs ”conventional” | 1.07 (0.28 to 4.69) | 0.45 (0.18 to 0.94) | 0.74 (0.46 to 1.15) |
| ⊕OOO | ⊕⊕⊕O | ⊕OOO |
| BB vs placebo | 0.97 (0.86 to 1.10) | 0.93 (0.77 to 1.13) | 0.73 (0.57 to 0.90) |
| ⊕⊕⊕O | ⊕⊕⊕O | ⊕⊕⊕O |
| ACE vs CCB | 1.02 (0.95 to 1.10) | 0.96 (0.83 to 1.07) | 1.19 (1.03 to 1.38) |
| ⊕⊕⊕O | ⊕⊕⊕O | ⊕⊕⊕O |
| ACE vs alpha-blockers | 0.98 (0.85 to 1.14) | 0.99 (0.77 to 1.27) | 0.91 (0.67 to 1.24) |
| ⊕⊕OO | ⊕OOO | ⊕OOO |
| ACE vs ARB | 1.02 (0.91 to 1.14) | 0.84 (0.68 to 1.04) | 1.08 (0.86 to 1.37) |
| ⊕⊕OO | ⊕OOO | ⊕OOO |
| ACE vs diuretics and/or BB | 1.06 (0.97 to 1.16) | 0.97 (0.83 to 1.12) | 1.10 (0.94 to 1.31) |
| ⊕⊕⊕O | ⊕⊕⊕O | ⊕OOO |
| ACE vs ”conventional” | 0.95 (0.25 to 4.14) | 0.37 (0.15 to 0.77) | 0.65 (0.41 to 1.05) |
| ⊕OOO | ⊕⊕⊕O | ⊕OOO |
| ACE vs placebo | 0.87 (0.79 to 0.96) | 0.76 (0.63 to 0.92) | 0.65 (0.53 to 0.78) |
| ⊕⊕⊕O | ⊕⊕OO | ⊕⊕⊕O |
| CCB vs alpha-blockers | 0.96 (0.83 to 1.11) | 1.03 (0.82 to 1.34) | 0.77 (0.57 to 1.04) |
| ⊕⊕OO | ⊕OOO | ⊕OOO |
| CCB vs ARB | 1.00 (0.91 to 1.10) | 0.87 (0.74 to 1.06) | 0.91 (0.75 to 1.11) |
| ⊕⊕⊕⊕ | ⊕⊕⊕O | ⊕⊕⊕O |
| CCB vs diuretics and/or BB | 1.04 (0.95 to 1.13) | 1.22 (0.89 to 6.63) | 0.93 (0.81 to 1.08) |
| ⊕⊕⊕O | ⊕OOO | ⊕⊕⊕O |
| CCB vs ”conventional” | 0.93 (0.24 to 4.01) | 0.39 (0.16 to 0.80) | 0.55 (0.35 to 0.87) |
| ⊕OOO | ⊕⊕⊕O | ⊕⊕OO |
| CCB vs placebo | 0.85 (0.78 to 0.93) | 0.80 (0.67 to 0.95) | 0.55 (0.46 to 0.64) |
| ⊕⊕⊕⊕ | ⊕⊕⊕⊕ | ⊕⊕⊕⊕ |
| Alpha-blockers vs ARB | 1.04 (0.88 to 1.23) | 0.84 (0.63 to 1.14) | 1.20 (0.85 to 1.69) |
| ⊕⊕OO | ⊕OOO | ⊕OOO |
| Alpha-blockers vs diuretics and/or BB | 1.09 (0.92 to 1.27) | 0.98 (0.74 to 1.28) | 1.21 (0.88 to 1.68) |
| ⊕OOO | ⊕OOO | ⊕OOO |
| Alpha-blockers vs ”conventional” | 0.97 (0.25 to 4.23) | 0.38 (0.15 to 0.79)  | 0.73 (0.43 to 1.23) |
| ⊕OOO | ⊕⊕⊕O | ⊕OOO |
| Alpha-blockers vs placebo | 0.89 (0.77 to 1.03) | 0.82 (0.59 to 1.00) | 0.72 (0.52 to 0.96)  |
| ⊕⊕OO | ⊕⊕OO | ⊕⊕OO |
| ARB vs diuretics and/or BB | 1.04 (0.92 to 1.18) | 1.16 (0.92 to 1.43) | 1.02 (0.80 to 1.29) |
| ⊕⊕OO | ⊕OOO | ⊕OOO |
| ARB vs ”conventional” | 0.94 (0.24 to 4.06) | 0.45 (0.18 to 0.82) | 0.60 (0.40 to 0.90) |
| ⊕OOO | ⊕⊕⊕O | ⊕⊕⊕O |
| ARB vs placebo | 0.85 (0.76 to 0.96) | 0.91 (0.72 to 1.14) | 0.60 (0.47 to 0.75) |
| ⊕⊕OO | ⊕OOO | ⊕⊕OO |
| Diuretics and/or BB vs ”conventional” | 0.90 (0.23 to 3.92) | 0.39 (0.16 to 0.80) | 0.59 (0.37 to 0.95) |
| ⊕OOO | ⊕⊕⊕O | ⊕⊕OO |
| Diuretics and/or BB vs placebo | 0.82 (0.73 to 0.92) | 0.79 (0.64 to 0.97) | 0.59 (0.48 to 0.72) |
| ⊕⊕⊕⊕ | ⊕⊕⊕⊕ | ⊕⊕⊕⊕ |
| ”Conventional” vs placebo | 0.91 (0.21 to 3.45) | 2.04 (1.00 to 5.13) | 1.00 (0.62 to 1.57) |
| ⊕OOO | ⊕⊕⊕O | ⊕OOO |

BB=Beta-blockers; ACE=Angiotensin Converting Enzyme Inhibitors; CCB=Calcium Channel Blockers; ARB=Angiotensin Receptor Blockers; ⊕⊕⊕⊕=High quality evidence; ⊕⊕⊕O=Moderate quality evidence; ⊕⊕OO=Low quality evidence; ⊕OOO=Very low quality evidence
