# Supplementary material for: Comparative effectiveness of antihypertensive medication for primary prevention of cardiovascular disease: systematic review and multiple treatments meta-analysis
Source: BMC Med. 2012 Apr 5;10:33. doi: 10.1186/1741-7015-10-33 (PMC3354999; doi:10.1186/1741-7015-10-33)
Supplement: Additional file 4 — Main findings (secondary outcomes). Full, comprehensive version of Table 4. [file 1741-7015-10-33-S4.DOC]

**Additional file 4 (Fretheim et al 2012).**

**Main findings (secondary** outcomes).

|  | **Angina** | **Heart failure** | **Diabetes incidence** |
| --- | --- | --- | --- |
| Diuretics vs BB | 0.96 (0.28 to 5.78) | 0.73 (0.54 to 0.96) | 1.09 (0.80 to 1.44) |
| ⊕OOO | ⊕⊕⊕O | ⊕⊕OO |
| Diuretics vs ACE | 0.97 (0.42 to 2.51) | 0.88 (0.76 to 1.06) | 1.43 (1.12 to 1.83) |
| ⊕⊕OO | ⊕⊕OO | ⊕⊕⊕O |
| Diuretics vs CCB | 1.05 (0.56 to 2.19) | 0.73 (0.62 to 0.84) | 1.27 (1.05 to 1.57) |
| ⊕⊕OO | ⊕⊕⊕O | ⊕⊕⊕⊕ |
| Diuretics vs alpha-blockers | 0.89 (0.31 to 2.52) | 0.51 (0.40 to 0.64) | - |
| ⊕OOO | ⊕⊕⊕O |  |
| Diuretics vs ARB | 0.86 (0.39 to 3.27) | 0.80 (0.61 to 0.98) | 1.59 (1.23 to 2.12) |
| ⊕OOO | ⊕⊕OO | ⊕⊕OO |
| Diuretics vs diuretics and/or BB | 1.07 (0.41 to 3.07) | 0.85 (0.71 to 1.06) | 1.23 (0.94 to 1.62) |
| ⊕OOO | ⊕OOO | ⊕OOO |
| Diuretics vs ”conventional” | - | 0.69 (0.39 to 1.11) | - |
|  | ⊕OOO |  |
| Diuretics vs placebo | 1.57 (0.18 to 16.52) | 0.46 (0.36 to 0.56) | - |
| ⊕OOO | ⊕⊕⊕⊕ |  |
| BB vs ACE | 1.03 (0.17 to 3.76) | 1.21 (0.91 to 1.69) | 1.31 (0.95 to 1.88) |
| ⊕OOO | ⊕OOO | ⊕OOO |
| BB vs CCB | 1.10 (0.23 to 3.31) | 1.00 (0.76 to 1.33) | 1.17 (0.89 to 1.61) |
| ⊕OOO | ⊕OOO | ⊕OOO |
| BB vs alpha-blockers | 0.93 (0.11 to 4.35) | 0.69 (0.50 to 1.02) | - |
| ⊕OOO | ⊕OOO |  |
| BB vs ARB | 0.88 (0.31 to 2.58) | 1.08 (0.86 to 1.38) | 1.46 (1.15 to 1.98) |
| ⊕⊕OO | ⊕⊕⊕⊕ | ⊕⊕⊕⊕ |
| BB vs diuretics and/or BB | 1.12 (0.18 to 4.24) | 1.17 (0.86 to 1.65) | 1.12 (0.81 to 1.64) |
| ⊕OOO | ⊕OOO | ⊕OOO |
| BB vs ”conventional” | - | 0.94 (0.54 to 1.56) | - |
|  | ⊕OOO |  |
| BB vs placebo | 1.52 (0.10 to 21.87) | 0.63 (0.45 to 0.86) | - |
| ⊕OOO | ⊕⊕⊕O |  |
| ACE vs CCB | 1.08 (0.48 to 2.44) | 0.82 (0.69 to 0.94) | 0.89 (0.73 to 1.10) |
| ⊕OOO | ⊕⊕⊕O | ⊕⊕OO |
| ACE vs alpha-blockers | 0.91 (0.22 to 3.42) | 0.58 (0.43 to 0.75) | - |
| ⊕OOO | ⊕⊕OO |  |
| ACE vs ARB | 0.86 (0.35 to 3.50) | 0.90 (0.67 to 1.10) | 1.11 (0.85 to 1.51) |
| ⊕OOO | ⊕OOO | ⊕OOO |
| ACE vs diuretics and/or BB | 1.10 (0.47 to 2.55) | 0.96 (0.81 to 1.15) | 0.86 (0.70 to 1.06) |
| ⊕OOO | ⊕⊕⊕O | ⊕⊕OO |
| ACE vs ”conventional” | - | 0.78 (0.43 to 1.25) | - |
|  | ⊕OOO |  |
| ACE vs placebo | 1.57 (0.16 to 19.07) | 0.51 (0.39 to 0.65) | - |
| ⊕OOO | ⊕⊕OO |  |
| CCB vs alpha-blockers | 0.85 (0.23 to 2.78) | 0.70 (0.53 to 0.92) | - |
| ⊕OOO | ⊕⊕OO |  |
| CCB vs ARB | 0.81 (0.45 to 2.30) | 1.10 (0.87 to 1.31) | 1.25 (1.02 to 1.56) |
| ⊕⊕OO | ⊕⊕⊕O | ⊕⊕⊕⊕ |
| CCB vs diuretics and/or BB | 1.02 (0.43 to 2.37) | 1.17 (1.01 to 1.40) | 0.96 (0.78 to 1.19) |
| ⊕⊕OO | ⊕⊕⊕O | ⊕⊕⊕O |
| CCB vs ”conventional” | - | 0.95 (0.54 to 1.51) | - |
|  | ⊕OOO |  |
| CCB vs placebo | 1.45 (0.16 to 16.44) | 0.63 (0.49 to 0.78) | - |
| ⊕OOO | ⊕⊕⊕⊕ |  |
| Alpha-blockers vs ARB | 0.95 (0.29 to 5.71) | 1.57 (1.09 to 2.12) | - |
| ⊕OOO | ⊕⊕OO |  |
| Alpha-blockers vs diuretics and/or BB | 1.20 (0.30 to 5.46) | 1.67 (1.26 to 2.31) | - |
| ⊕OOO | ⊕⊕OO |  |
| Alpha-blockers vs ”conventional” | - | 1.36 (0.72 to 2.32) | - |
|  | ⊕OOO |  |
| Alpha-blockers vs placebo | 1.77 (0.17 to 22.73) | 0.90 (0.64 to 1.21) | - |
| ⊕OOO | ⊕OOO |  |
| ARB vs diuretics and/or BB | 1.27 (0.30 to 3.20) | 1.07 (0.86 to 1.45) | 0.77 (0.57 to 1.03) |
| ⊕OOO | ⊕OOO | ⊕OOO |
| ARB vs ”conventional” | - | 0.86 (0.52 to 1.36) | - |
|  | ⊕OOO |  |
| ARB vs placebo | 1.70 (0.15 to 20.46) | 0.57 (0.44 to 0.77) | - |
| ⊕OOO | ⊕⊕OO |  |
| Diuretics and/or BB vs ”conventional” | - | 0.81 (0.44 to 1.31) | - |
|  | ⊕OOO |  |
| Diuretics and/or BB vs placebo | 1.43 (0.14 to 18.07) | 0.53 (0.40 to 0.68) | - |
| ⊕OOO | ⊕⊕OO |  |
| ”Conventional” vs placebo | - | 0.66 (0.39 to 1.18) | - |
|  | ⊕OOO |  |

BB=Beta-blockers; ACE=Angiotensin Converting Enzyme Inhibitors; CCB=Calcium Channel Blockers; ARB=Angiotensin Receptor Blockers; ⊕⊕⊕⊕=High quality evidence; ⊕⊕⊕O=Moderate quality evidence; ⊕⊕OO=Low quality evidence; ⊕OOO=Very low quality evidence
