# Supplementary material for: Comparative effectiveness of antihypertensive medication for primary prevention of cardiovascular disease: systematic review and multiple treatments meta-analysis
Source: BMC Med. 2012 Apr 5;10:33. doi: 10.1186/1741-7015-10-33 (PMC3354999; doi:10.1186/1741-7015-10-33)
Supplement: Additional file 5 — GRADE-profiles. In this file, the reasoning for our grading of the evidence is presented, for all outcomes across all comparisons. [file 1741-7015-10-33-S5.DOC]

# Additional file 5 (Fretheim et al 2012):

# GRADE-profiles

[Diuretics vs beta-blockers 3](#__RefHeading___Toc297192198)

[Diuretics vs ACE-inhibitors 5](#__RefHeading___Toc297192199)

[Diuretics vs CCBs 7](#__RefHeading___Toc297192200)

[Diuretics vs alpha-blockers 9](#__RefHeading___Toc297192201)

[Diuretics vs ARBs 11](#__RefHeading___Toc297192202)

[Diuretics vs diuretics and/or beta-blockers 13](#__RefHeading___Toc297192203)

[Diuretics vs "conventional drugs" 15](#__RefHeading___Toc297192204)

[Diuretics vs placebo/control 16](#__RefHeading___Toc297192205)

[Beta-blockers vs ACE-inhibitors 18](#__RefHeading___Toc297192206)

[Beta-blockers vs CCBs 20](#__RefHeading___Toc297192207)

[Beta-blockers vs alpha-blockers 22](#__RefHeading___Toc297192208)

[Beta-blockers vs ARBs 24](#__RefHeading___Toc297192209)

[Beta-blockers vs diuretics and/or beta-blockers 26](#__RefHeading___Toc297192210)

[Beta-blockers vs "conventional drugs" 28](#__RefHeading___Toc297192211)

[Beta-blockers vs placebo/control 29](#__RefHeading___Toc297192212)

[ACE-inhibitors vs CCBs 31](#__RefHeading___Toc297192213)

[ACE-inhibitors vs alpha-blockers 33](#__RefHeading___Toc297192214)

[ACE-inhibitors vs ARBs 35](#__RefHeading___Toc297192215)

[ACE-inhibitors vs diuretics and/or beta-blockers 37](#__RefHeading___Toc297192216)

[ACE-inhibitors vs "conventional drugs" 39](#__RefHeading___Toc297192217)

[ACE-inhibitors vs placebo/control 40](#__RefHeading___Toc297192218)

[CCBs vs alpha-blockers 42](#__RefHeading___Toc297192219)

[CCBs vs ARBs 44](#__RefHeading___Toc297192220)

[CCBs vs diuretics and/or beta-blockers 46](#__RefHeading___Toc297192221)

[CCBs vs "conventional drugs" 48](#__RefHeading___Toc297192222)

[CCBs vs placebo/control 49](#__RefHeading___Toc297192223)

[Alpha-blockers vs ARBs 51](#__RefHeading___Toc297192224)

[Alpha-blockers vs diuretics and/or beta-blockers 53](#__RefHeading___Toc297192225)

[Alpha-blockers vs "conventional drugs" 55](#__RefHeading___Toc297192226)

[Alpha-blockers vs placebo/control 56](#__RefHeading___Toc297192227)

[ARBs vs diuretics and/or beta-blockers 58](#__RefHeading___Toc297192228)

[ARBs vs "conventional drugs" 60](#__RefHeading___Toc297192229)

[ARBs vs placebo/control 61](#__RefHeading___Toc297192230)

[Diuretics and/or beta-blockers vs "conventional drugs" 63](#__RefHeading___Toc297192231)

[Diuretics and/or beta-blockers vs placebo/control 64](#__RefHeading___Toc297192232)

["Conventional drugs" vs placebo/control 66](#__RefHeading___Toc297192233)

Diuretics vs beta-blockers

**Author(s): A Fretheim**
**Date:** 2011-05-31
**Question:** Should Diuretics vs beta-blockers be used for hypertension?

| **Quality assessment** | | | | | | | **No of patients** | | **Effect** | | **Quality** | **Importance** |
| --- | --- | --- | --- | --- | --- | --- | --- | --- | --- | --- | --- | --- |
|  |  |  |  |  |  |  |  |  |  |  |  |  |
| **No of studies (direct comparisons)** | **Design** | **Risk of bias** | **Inconsistency** | **Indirectness** | **Imprecision** | **Other considerations** | **Diuretics** | **Beta-blockers** | **Relative (95% CI)** | **Absolute** |  |  |
| **Total mortality** | | | | | | | | | | | | |
| 2 | randomised trials | serious^1^ | no serious inconsistency | no serious indirectness | no serious imprecision | none | - | - | RR 0.90 (0.8 to 1.01) | - | ⊕⊕⊕O MODERATE |  |
|  |  |  |  |  |  |  |  | 0% |  | - |  |  |
| **Myocardial infarction** | | | | | | | | | | | | |
| 2 | randomised trials | serious^1^ | serious^2^ | no serious indirectness | no serious imprecision | none | - | - | RR 0.82 (0.68 to 0.98) | - | ⊕⊕OO LOW |  |
|  |  |  |  |  |  |  |  | 0% |  | - |  |  |
| **Stroke** | | | | | | | | | | | | |
| 2 | randomised trials | serious^1^ | no serious inconsistency | no serious indirectness | serious^3^ | none | - | - | RR 0.83 (0.68 to 1.07) | - | ⊕⊕OO LOW |  |
|  |  |  |  |  |  |  |  | 0% |  | - |  |  |
| **Angina** | | | | | | | | | | | | |
| 0 | only indirect comparisons |  |  |  | Serious^5^ | none | - | - | RR 0.96 (0.28 to 5.78) | - | ⊕OOO VERY LOW |  |
|  |  |  |  |  |  |  |  | 0% |  | - |  |  |
| **Heart failure** | | | | | | | | | | | | |
| 1 | randomised trials | serious^4^ | no serious inconsistency | no serious indirectness | no serious imprecision | none | - | - | RR 0.73 (0.54 to 0.96) | - | ⊕⊕⊕O MODERATE |  |
|  |  |  |  |  |  |  |  | 0% |  | - |  |  |
| **Diabetes** | | | | | | | | | | | | |
| 1 | randomised trials | serious^4^ | no serious inconsistency | no serious indirectness | serious^3^ | none | - | - | RR 1.09 (0.8 to 1.44) | - | ⊕⊕OO LOW |  |
|  |  |  |  |  |  |  |  | 0% |  | - |  |  |

^1^ Both studies rated "moderate quality" by expert group.
^2^ I2-squared=66%
^3^ Wide credibility interval, including both no difference and important difference.
^4^ Study rated "moderate quality" by expert group.
^5^ Wide credibility interval, including important differences in both directions.

Diuretics vs ACE-inhibitors

**Author(s): A Fretheim**
**Date:** 2011-05-31
**Question:** Should Diuretics vs ACE-inhibitors be used for hypertension?

| **Quality assessment** | | | | | | | **No of patients** | | **Effect** | | **Quality** | **Importance** |
| --- | --- | --- | --- | --- | --- | --- | --- | --- | --- | --- | --- | --- |
|  |  |  |  |  |  |  |  |  |  |  |  |  |
| **No of studies (direct comparisons)** | **Design** | **Risk of bias** | **Inconsistency** | **Indirectness** | **Imprecision** | **Other considerations** | **Diuretics** | **ACE-inhibitors** | **Relative (95% CI)** | **Absolute** |  |  |
| **Total mortality** | | | | | | | | | | | | |
| 3 | randomised trials | serious^1^ | no serious inconsistency | no serious indirectness | no serious imprecision | none | - | - | RR 1.00 (0.93 to 1.08) | - | ⊕⊕⊕O MODERATE |  |
|  |  |  |  |  |  |  |  | 0% |  | - |  |  |
| **Myocardial infarction** | | | | | | | | | | | | |
| 2 | randomised trials | serious^2^ | no serious inconsistency | no serious indirectness | no serious imprecision | none | - | - | RR 1.00 (0.88 to 1.15) | - | ⊕⊕⊕O MODERATE |  |
|  |  |  |  |  |  |  |  | 0% |  | - |  |  |
| **Stroke** | | | | | | | | | | | | |
| 3 | randomised trials | serious^1^ | no serious inconsistency | no serious indirectness | no serious imprecision | none | - | - | RR 0.94 (0.81 to 1.1) | - | ⊕⊕⊕O MODERATE |  |
|  |  |  |  |  |  |  |  | 0% |  | - |  |  |
| **Angina** | | | | | | | | | | | | |
| 1 | randomised trials | serious^3^ | no serious inconsistency | no serious indirectness | serious^4^ | none | - | - | RR 0.97 (0.42 to 2.51) | - | ⊕⊕OO LOW |  |
|  |  |  |  |  |  |  |  | 0% |  | - |  |  |
| **Heart failure** | | | | | | | | | | | | |
| 2 | randomised trials | serious^2^ | serious^5^ | no serious indirectness | no serious imprecision | none | - | - | RR 0.88 (0.76 to 1.06) | - | ⊕⊕OO LOW |  |
|  |  |  |  |  |  |  |  | 0% |  | - |  |  |
| **Diabetes** | | | | | | | | | | | | |
| 1 | randomised trials | serious^6^ | no serious inconsistency | no serious indirectness | no serious imprecision | none | - | - | RR 1.43 (1.12 to 1.82) | - | ⊕⊕⊕O MODERATE |  |
|  |  |  |  |  |  |  |  | 0% |  | - |  |  |

^1^ All trials rated "moderate quality" by expert group.
^2^ Both trials rated "moderate quality" by expert group.
^3^ Trial rated "moderate quality" by expert group.
^4^ Wide credibility interval, including both no difference and important difference.
^5^ I-squared=66%
^6^ No explanation was provided

Diuretics vs CCBs

**Author(s): A Fretheim**
**Date:** 2011-05-31
**Question:** Should Diuretics vs CCBs be used for hypertension?

| **Quality assessment** | | | | | | | **No of patients** | | **Effect** | | **Quality** | **Importance** |
| --- | --- | --- | --- | --- | --- | --- | --- | --- | --- | --- | --- | --- |
|  |  |  |  |  |  |  |  |  |  |  |  |  |
| **No of studies (direct comparisons)** | **Design** | **Risk of bias** | **Inconsistency** | **Indirectness** | **Imprecision** | **Other considerations** | **Diuretics** | **CCBs** | **Relative (95% CI)** | **Absolute** |  |  |
| **Total mortality** | | | | | | | | | | | | |
| 4 | randomised trials | serious^1^ | no serious inconsistency | no serious indirectness | no serious imprecision | none | - | - | RR 1.03 (0.96 to 1.1) | - | ⊕⊕⊕O MODERATE |  |
|  |  |  |  |  |  |  |  | 0% |  | - |  |  |
| **Myocardial infarction** | | | | | | | | | | | | |
| 4 | randomised trials | serious^1^ | no serious inconsistency | no serious indirectness | no serious imprecision | none | - | - | RR 0.96 (0.84 to 1.07) | - | ⊕⊕⊕O MODERATE |  |
|  |  |  |  |  |  |  |  | 0% |  | - |  |  |
| **Stroke** | | | | | | | | | | | | |
| 4 | randomised trials | serious^1^ | no serious inconsistency | no serious indirectness | serious^2^ | none | - | - | RR 1.12 (0.97 to 1.29) | - | ⊕⊕OO LOW |  |
|  |  |  |  |  |  |  |  | 0% |  | - |  |  |
| **Angina** | | | | | | | | | | | | |
| 3 | randomised trials | no serious risk of bias | no serious inconsistency | no serious indirectness | very serious^2,3^ | none | - | - | RR 1.05 (0.56 to 2.19) | - | ⊕⊕OO LOW |  |
|  |  |  |  |  |  |  |  | 0% |  | - |  |  |
| **Heart failure** | | | | | | | | | | | | |
| 4 | randomised trials | serious^1^ | no serious inconsistency | no serious indirectness | no serious imprecision | none | - | - | RR 0.73 (0.62 to 0.84) | - | ⊕⊕⊕O MODERATE |  |
|  |  |  |  |  |  |  |  | 0% |  | - |  |  |
| **Diabetes** | | | | | | | | | | | | |
| 3 | randomised trials | no serious risk of bias | no serious inconsistency | no serious indirectness | no serious imprecision | none | - | - | RR 1.27 (1.05 to 1.57) | - | ⊕⊕⊕⊕ HIGH |  |
|  |  |  |  |  |  |  |  | 0% |  | - |  |  |

^1^ 2 of 4 studies rated "moderate quality" by expert group.
^2^ Wide credibility interval, including both no difference and important difference.
^3^ Wide credibility interval, including important differences in opposite directions.

Diuretics vs alpha-blockers

**Author(s): A Fretheim**
**Date:** 2011-05-31
**Question:** Should Diuretics vs alpha-blockers be used for hypertension?

| **Quality assessment** | | | | | | | **No of patients** | | **Effect** | | **Quality** | **Importance** |
| --- | --- | --- | --- | --- | --- | --- | --- | --- | --- | --- | --- | --- |
|  |  |  |  |  |  |  |  |  |  |  |  |  |
| **No of studies (direct comparisons)** | **Design** | **Risk of bias** | **Inconsistency** | **Indirectness** | **Imprecision** | **Other considerations** | **Diuretics** | **Alpha-blockers** | **Relative (95% CI)** | **Absolute** |  |  |
| **Total mortality** | | | | | | | | | | | | |
| 1 | randomised trials | serious^1^ | no serious inconsistency | no serious indirectness | no serious imprecision | none | - | - | RR 0.98 (0.87 to 1.12) | - | ⊕⊕⊕O MODERATE |  |
|  |  |  |  |  |  |  |  | 0% |  | - |  |  |
| **Myocardial infarction** | | | | | | | | | | | | |
| 1 | randomised trials | serious^1^ | no serious inconsistency | no serious indirectness | no serious imprecision | none | - | - | RR 0.99 (0.8 to 1.23) | - | ⊕⊕⊕O MODERATE |  |
|  |  |  |  |  |  |  |  | 0% |  | - |  |  |
| **Stroke** | | | | | | | | | | | | |
| 1 | randomised trials | serious^1^ | no serious inconsistency | no serious indirectness | serious^2^ | none | - | - | RR 0.85 (0.66 to 1.12) | - | ⊕⊕OO LOW |  |
|  |  |  |  |  |  |  |  | 0% |  | - |  |  |
| **Angina** | | | | | | | | | | | | |
| 1 | randomised trials | serious^1^ | no serious inconsistency | no serious indirectness | very serious^3^ | none | - | - | RR 0.89 (0.31 to 2.52) | - | ⊕OOO VERY LOW |  |
|  |  |  |  |  |  |  |  | 0% |  | - |  |  |
| **Heart failure** | | | | | | | | | | | | |
| 1 | randomised trials | serious^1^ | no serious inconsistency | no serious indirectness | no serious imprecision | none | - | - | RR 0.51 (0.41 to 0.64) | - | ⊕⊕⊕O MODERATE |  |
|  |  |  |  |  |  |  |  | 0% |  | - |  |  |

^1^ Study rated "moderate quality" by expert group.
^2^ Wide credibility interval, including both no difference and important difference.
^3^ Wide credibility interval, including important differences in opposite directions.

Diuretics vs ARBs

**Author(s): A Fretheim**
**Date:** 2011-05-31
**Question:** Should Diuretics vs ARBs be used for hypertension?

| **Quality assessment** | | | | | | | **No of patients** | | **Effect** | | **Quality** | **Importance** |
| --- | --- | --- | --- | --- | --- | --- | --- | --- | --- | --- | --- | --- |
|  |  |  |  |  |  |  |  |  |  |  |  |  |
| **No of studies (direct comparisons)** | **Design** | **Risk of bias** | **Inconsistency** | **Indirectness** | **Imprecision** | **Other considerations** | **Diuretics** | **ARBs** | **Relative (95% CI)** | **Absolute** |  |  |
| **Total moratlity** | | | | | | | | | | | | |
| 0 | only indirect comparisons |  |  |  | no serious imprecision | none | - | - | RR 1.02 (0.92 to 1.14) | - | ⊕⊕OO LOW |  |
|  |  |  |  |  |  |  |  | 0% |  | - |  |  |
| **Myocardial infarction** | | | | | | | | | | | | |
| 0 | only indirect comparisons |  |  |  | serious^1^ | none | - | - | RR 0.83 (0.69 to 1.03) | - | ⊕OOO VERY LOW |  |
|  |  |  |  |  |  |  |  | 0% |  | - |  |  |
| **Stroke** | | | | | | | | | | | | |
| 0 | only indirect comparisons |  |  |  | serious^1^ | none | - | - | RR 1.02 (0.82 to 1.28) | - | ⊕OOO VERY LOW |  |
|  |  |  |  |  |  |  |  | 0% |  | - |  |  |
| **Angina** | | | | | | | | | | | | |
| 0 | only indirect comparisons |  |  |  | very serious^2^ | none | - | - | RR 0.86 (0.39 to 3.27) | - | ⊕OOO VERY LOW |  |
|  |  |  |  |  |  |  |  | 0% |  | - |  |  |
| **Heart failure** | | | | | | | | | | | | |
| 0 | only indirect comparisons |  |  |  | no serious imprecision | none | - | - | RR 0.80 (0.61 to 0.98) | - | ⊕⊕OO LOW |  |
|  |  |  |  |  |  |  |  | 0% |  | - |  |  |
| **Diabetes** | | | | | | | | | | | | |
| 0 | only indirect comparisons |  |  |  | no serious imprecision | none | - | - | RR 1.59 (1.23 to 2.12) | - | ⊕⊕OO LOW |  |
|  |  |  |  |  |  |  |  | 0% |  | - |  |  |

^1^ Wide credibility interval, including both no difference and important difference.
^2^ Wide credibility interval, including important differences in opposite directions.

Diuretics vs diuretics and/or beta-blockers

**Author(s): A Fretheim**
**Date:** 2011-05-31
**Question:** Should Diuretics vs diuretics and/or beta-blockers be used for hypertension?

| **Quality assessment** | | | | | | | **No of patients** | | **Effect** | | **Quality** | **Importance** |
| --- | --- | --- | --- | --- | --- | --- | --- | --- | --- | --- | --- | --- |
|  |  |  |  |  |  |  |  |  |  |  |  |  |
| **No of studies (direct comparisons)** | **Design** | **Risk of bias** | **Inconsistency** | **Indirectness** | **Imprecision** | **Other considerations** | **Diuretics** | **Diuretics and/or beta-blockers** | **Relative (95% CI)** | **Absolute** |  |  |
| **Total mortality** | | | | | | | | | | | | |
| 0 | only indirect comparisons |  |  |  | no serious imprecision | none | - | - | RR 1.07 (0.97 to 1.17) | - | ⊕⊕OO LOW |  |
|  |  |  |  |  |  |  |  | 0% |  | - |  |  |
| **Myocardial infarction** | | | | | | | | | | | | |
| 0 | only indirect comparisons |  |  |  | no serious imprecision | none | - | - | RR 0.97 (0.82 to 1.14) | - | ⊕⊕OO LOW |  |
|  |  |  |  |  |  |  |  | 0% |  | - |  |  |
| **Stroke** | | | | | | | | | | | | |
| 0 | only indirect comparisons |  |  |  | serious^1^ | none | - | - | RR 1.04 (0.87 to 1.25) | - | ⊕OOO VERY LOW |  |
|  |  |  |  |  |  |  |  | 0% |  | - |  |  |
| **Angina** | | | | | | | | | | | | |
| 0 | only indirect comparisons |  |  |  | very serious^2^ | none | - | - | RR 1.07 (0.41 to 3.07) | - | ⊕OOO VERY LOW |  |
|  |  |  |  |  |  |  |  | 0% |  | - |  |  |
| **Heart failure** | | | | | | | | | | | | |
| 0 | only indirect comparisons |  |  |  | serious^1^ | none | - | - | RR 0.85 (0.71 to 1.06) | - | ⊕OOO VERY LOW |  |
|  |  |  |  |  |  |  |  | 0% |  | - |  |  |
| **Diabetes** | | | | | | | | | | | | |
| 0 | only indirect comparisons |  |  |  | serious^1^ | none | - | - | RR 1.23 (0.94 to 1.62) | - | ⊕OOO VERY LOW |  |
|  |  |  |  |  |  |  |  | 0% |  | - |  |  |

^1^ Wide credibility interval, including both no difference and important difference.
^2^ Wide credibility interval, including important differences in opposite directions.

Diuretics vs "conventional drugs"

**Author(s): A Fretheim**
**Date:** 2011-05-31
**Question:** Should Diuretics vs "conventional drugs" be used for hypertension?

| **Quality assessment** | | | | | | | **No of patients** | | **Effect** | | **Quality** | **Importance** |
| --- | --- | --- | --- | --- | --- | --- | --- | --- | --- | --- | --- | --- |
|  |  |  |  |  |  |  |  |  |  |  |  |  |
| **No of studies (direct comparisons)** | **Design** | **Risk of bias** | **Inconsistency** | **Indirectness** | **Imprecision** | **Other considerations** | **Diuretics** | **"conventional drugs"** | **Relative (95% CI)** | **Absolute** |  |  |
| **Total mortality** | | | | | | | | | | | | |
| 0 | only indirect comparisons |  |  |  | very serious^1^ | none | - | - | RR 0.96 (0.25 to 4.12) | - | ⊕OOO VERY LOW |  |
|  |  |  |  |  |  |  |  | 0% |  | - |  |  |
| **Myocardial infarction** | | | | | | | | | | | | |
| 0 | only indirect comparisons |  |  |  | no serious imprecision | large effect^2^ | - | - | RR 0.37 (0.15 to 0.77) | - | ⊕⊕⊕O MODERATE |  |
|  |  |  |  |  |  |  |  | 0% |  | - |  |  |
| **Stroke** | | | | | | | | | | | | |
| 0 | only indirect comparisons |  |  |  | no serious imprecision | none | - | - | RR 0.61 (0.39 to 0.98) | - | ⊕⊕OO LOW |  |
|  |  |  |  |  |  |  |  | 0% |  | - |  |  |
| **Heart failure** | | | | | | | | | | | | |
| 0 | only indirect comparisons |  |  |  | serious^3^ | none | - | - | RR 0.69 (0.39 to 1.11) | - | ⊕OOO VERY LOW |  |
|  |  |  |  |  |  |  |  | 0% |  | - |  |  |

^1^ Wide credibility interval, including important differences in opposite directions.

^2^ RR<0.5

^3^ Wide credibility interval, including important differences in opposite directions.

Diuretics vs placebo/control

**Author(s): A Fretheim**
**Date:** 2011-05-31
**Question:** Should Diuretics vs placebo/control be used for hypertension?

| **Quality assessment** | | | | | | | **No of patients** | | **Effect** | | **Quality** | **Importance** |
| --- | --- | --- | --- | --- | --- | --- | --- | --- | --- | --- | --- | --- |
|  |  |  |  |  |  |  |  |  |  |  |  |  |
| **No of studies (direct comparisons)** | **Design** | **Risk of bias** | **Inconsistency** | **Indirectness** | **Imprecision** | **Other considerations** | **Diuretics** | **Placebo/control** | **Relative (95% CI)** | **Absolute** |  |  |
| **Total mortality** | | | | | | | | | | | | |
| 7 | randomised trials | no serious risk of bias | no serious inconsistency | no serious indirectness | no serious imprecision | none | - | - | RR 0.88 (0.8 to 0.95) | - | ⊕⊕⊕⊕ HIGH |  |
|  |  |  |  |  |  |  |  | 0% |  | - |  |  |
| **Myocardial infarction** | | | | | | | | | | | | |
| 6 | randomised trials | no serious risk of bias | no serious inconsistency | no serious indirectness | no serious imprecision | none | - | - | RR 0.76 (0.65 to 0.89) | - | ⊕⊕⊕⊕ HIGH |  |
|  |  |  |  |  |  |  |  | 0% |  | - |  |  |
| **Stroke** | | | | | | | | | | | | |
| 7 | randomised trials | no serious risk of bias | no serious inconsistency | no serious indirectness | no serious imprecision | none | - | - | RR 0.61 (0.52 to 0.71) | - | ⊕⊕⊕⊕ HIGH |  |
|  |  |  |  |  |  |  |  | 0% |  | - |  |  |
| **Angina** | | | | | | | | | | | | |
| 1 | randomised trials | serious^1^ | no serious inconsistency | no serious indirectness | very serious^2^ | none | - | - | RR 1.57 (0.18 to 16.52) | - | ⊕OOO VERY LOW |  |
|  |  |  |  |  |  |  |  | 0% |  | - |  |  |
| **Heart failure** | | | | | | | | | | | | |
| 4 | randomised trials | no serious risk of bias | no serious inconsistency | no serious indirectness | no serious imprecision | none | - | - | RR 0.46 (0.36 to 0.56) | - | ⊕⊕⊕⊕ HIGH |  |
|  |  |  |  |  |  |  |  | 0% |  | - |  |  |

^1^ Study assessed as "moderate quality" by expert group.
^2^ Wide credible interval, including important differences in opposite directions.

Beta-blockers vs ACE-inhibitors

**Author(s): A Fretheim**
**Date:** 2011-05-31
**Question:** Should Beta-blockers vs ACE-inhibitors be used for hypertension?

| **Quality assessment** | | | | | | | **No of patients** | | **Effect** | | **Quality** | **Importance** |
| --- | --- | --- | --- | --- | --- | --- | --- | --- | --- | --- | --- | --- |
|  |  |  |  |  |  |  |  |  |  |  |  |  |
| **No of studies (direct comparisons)** | **Design** | **Risk of bias** | **Inconsistency** | **Indirectness** | **Imprecision** | **Other considerations** | **Beta-blockers** | **ACE-inhibitors** | **Relative (95% CI)** | **Absolute** |  |  |
| **Total mortality** | | | | | | | | | | | | |
| 0 | only indirect comparisons |  |  |  | serious^1^ | none | - | - | RR 1.12 (0.98 to 1.27) | - | ⊕OOO VERY LOW |  |
|  |  |  |  |  |  |  |  | 0% |  | - |  |  |
| **Myocardial infarction** | | | | | | | | | | | | |
| 0 | only indirect comparisons |  |  |  | no serious imprecision | none | - | - | RR 1.22 (1 to 1.52) | - | ⊕⊕OO LOW |  |
|  |  |  |  |  |  |  |  | 0% |  | - |  |  |
| **Stroke** | | | | | | | | | | | | |
| 0 | only indirect comparisons |  |  |  | serious^1^ | none | - | - | RR 1.13 (0.86 to 1.42) | - | ⊕OOO VERY LOW |  |
|  |  |  |  |  |  |  |  | 0% |  | - |  |  |
| **Angina** | | | | | | | | | | | | |
| 0 | only indirect comparisons |  |  |  | very serious^2^ | none | - | - | RR 1.03 (017 to 3.76) | - | ⊕OOO VERY LOW |  |
|  |  |  |  |  |  |  |  | 0% |  | - |  |  |
| **Heart failure** | | | | | | | | | | | | |
| 0 | only indirect comparisons |  |  |  | serious^1^ | none | - | - | RR 1.21 (0.91 to 1.69) | - | ⊕OOO VERY LOW |  |
|  |  |  |  |  |  |  |  | 0% |  | - |  |  |
| **Diabetes** | | | | | | | | | | | | |
| 0 | only indirect comparisons |  |  |  | serious^1^ | none | - | - | RR 1.31 (0.95 to 1.88) | - | ⊕OOO VERY LOW |  |
|  |  |  |  |  |  |  |  | 0% |  | - |  |  |

^1^ Wide credibility interval, including both no difference and important difference.
^2^ Wide credibility interval, including important differences in opposite directions.

Beta-blockers vs CCBs

**Author(s): A Fretheim**
**Date:** 2011-05-31
**Question:** Should Beta-blockers vs CCBs be used for hypertension?

| **Quality assessment** | | | | | | | **No of patients** | | **Effect** | | **Quality** | **Importance** |
| --- | --- | --- | --- | --- | --- | --- | --- | --- | --- | --- | --- | --- |
|  |  |  |  |  |  |  |  |  |  |  |  |  |
| **No of studies (direct comparisons)** | **Design** | **Risk of bias** | **Inconsistency** | **Indirectness** | **Imprecision** | **Other considerations** | **Beta-blockers** | **CCBs** | **Relative (95% CI)** | **Absolute** |  |  |
| **Total mortality** | | | | | | | | | | | | |
| 0 | only indirect comparisons |  |  |  | no serious imprecision | none | - | - | RR 1.14 (1.01 to 1.28) | - | ⊕⊕OO LOW |  |
|  |  |  |  |  |  |  |  | 0% |  | - |  |  |
| **Myocardial infarction** | | | | | | | | | | | | |
| 0 | only indirect comparisons |  |  |  | serious^1^ | none | - | - | RR 1.17 (0.97 to 1.42) | - | ⊕OOO VERY LOW |  |
|  |  |  |  |  |  |  |  | 0% |  | - |  |  |
| **Stroke** | | | | | | | | | | | | |
| 0 | only indirect comparisons |  |  |  | no serious imprecision | none | - | - | RR 1.34 (1.05 to 1.64) | - | ⊕⊕OO LOW |  |
|  |  |  |  |  |  |  |  | 0% |  | - |  |  |
| **Angina** | | | | | | | | | | | | |
| 0 | only indirect comparisons |  |  |  | very serious^2^ | none | - | - | RR 1.10 (0.23 to 3.31) | - | ⊕OOO VERY LOW |  |
|  |  |  |  |  |  |  |  | 0% |  | - |  |  |
| **Heart failure** | | | | | | | | | | | | |
| 0 | only indirect comparisons |  |  |  | very serious^2^ | none | - | - | RR 1.00 (0.76 to 1.33) | - | ⊕OOO VERY LOW |  |
|  |  |  |  |  |  |  |  | 0% |  | - |  |  |
| **Diabetes** | | | | | | | | | | | | |
| 0 | only indirect comparisons |  |  |  | serious^1^ | none | - | - | RR 1.17 (0.89 to 1.61) | - | ⊕OOO VERY LOW |  |
|  |  |  |  |  |  |  |  | 0% |  | - |  |  |

^1^ Wide credibility interval, including both no difference and important difference.
^2^ Wide credibility interval, including important differences in opposite directions.

Beta-blockers vs alpha-blockers

**Author(s): A Fretheim**
**Date:** 2011-05-31
**Question:** Should Beta-blockers vs alpha-blockers be used for hypertension?

| **Quality assessment** | | | | | | | **No of patients** | | **Effect** | | **Quality** | **Importance** |
| --- | --- | --- | --- | --- | --- | --- | --- | --- | --- | --- | --- | --- |
|  |  |  |  |  |  |  |  |  |  |  |  |  |
| **No of studies (direct comparisons)** | **Design** | **Risk of bias** | **Inconsistency** | **Indirectness** | **Imprecision** | **Other considerations** | **Beta-blockers** | **Alpha-blockers** | **Relative (95% CI)** | **Absolute** |  |  |
| **Total mortality** | | | | | | | | | | | | |
| 0 | only indirect comparisons |  |  |  | serious^1^ | none | - | - | RR 1.09 (0.93 to 1.3) | - | ⊕OOO VERY LOW |  |
|  |  |  |  |  |  |  |  | 0% |  | - |  |  |
| **Myocardial infarction** | | | | | | | | | | | | |
| 0 | only indirect comparisons |  |  |  | serious^1^ | none | - | - | RR 1.20 (0.92 to 1.61) | - | ⊕OOO VERY LOW |  |
|  |  |  |  |  |  |  |  | 0% |  | - |  |  |
| **Stroke** | | | | | | | | | | | | |
| 0 | only indirect comparisons |  |  |  | very serious^2^ | none | - | - | RR 1.02 (0.71 to 1.42) | - | ⊕OOO VERY LOW |  |
|  |  |  |  |  |  |  |  | 0% |  | - |  |  |
| **Angina** | | | | | | | | | | | | |
| 0 | only indirect comparisons |  |  |  | very serious^2^ | none | - | - | RR 0.93 (0.11 to 4.35) | - | ⊕OOO VERY LOW |  |
|  |  |  |  |  |  |  |  | 0% |  | - |  |  |
| **Heart failure** | | | | | | | | | | | | |
| 0 | only indirect comparisons |  |  |  | serious^1^ | none | - | - | RR 0.69 (0.5 to 1.02) | - | ⊕OOO VERY LOW |  |
|  |  |  |  |  |  |  |  | 0% |  | - |  |  |

^1^ Wide credibility interval, including both no difference and important difference.
^2^ Wide credibility interval, including important differences in opposite directions.

Beta-blockers vs ARBs

**Author(s): A Fretheim**
**Date:** 2011-06-05
**Question:** Should Beta-blockers vs ARBs be used for hypertension?

| **Quality assessment** | | | | | | | **No of patients** | | **Effect** | | **Quality** | **Importance** |
| --- | --- | --- | --- | --- | --- | --- | --- | --- | --- | --- | --- | --- |
|  |  |  |  |  |  |  |  |  |  |  |  |  |
| **No of studies (direct comparisons)** | **Design** | **Risk of bias** | **Inconsistency** | **Indirectness** | **Imprecision** | **Other considerations** | **Beta-blockers** | **ARBs** | **Relative (95% CI)** | **Absolute** |  |  |
| **Total mortality** | | | | | | | | | | | | |
| 1 | randomised trials | no serious risk of bias | no serious inconsistency | no serious indirectness | no serious imprecision | none | - | - | RR 1.14 (1.02 to 1.28) | - | ⊕⊕⊕⊕ HIGH |  |
|  |  |  |  |  |  |  |  | 0% |  | - |  |  |
| **Myocardial infarction** | | | | | | | | | | | | |
| 1 | randomised trials | no serious risk of bias | no serious inconsistency | no serious indirectness | serious^1^ | none | - | - | RR 1.02 (0.84 to 1.27) | - | ⊕⊕⊕O MODERATE |  |
|  |  |  |  |  |  |  |  | 0% |  | - |  |  |
| **Stroke** | | | | | | | | | | | | |
| 1 | randomised trials | no serious risk of bias | no serious inconsistency | no serious indirectness | serious^1^ | none | - | - | RR 1.23 (0.96 to 1.49) | - | ⊕⊕⊕O MODERATE |  |
|  |  |  |  |  |  |  |  | 0% |  | - |  |  |
| **Angina** | | | | | | | | | | | | |
| 1 | randomised trials | no serious risk of bias | no serious inconsistency | no serious indirectness | very serious^2^ | none | - | - | RR 0.88 (0.31 to 2.58) | - | ⊕⊕OO LOW |  |
|  |  |  |  |  |  |  |  | 0% |  | - |  |  |
| **Heart failure** | | | | | | | | | | | | |
| 1 | randomised trials | no serious risk of bias | no serious inconsistency | no serious indirectness | no serious imprecision | none | - | - | RR 0.63 (0.45 to 0.86) | - | ⊕⊕⊕⊕ HIGH |  |
|  |  |  |  |  |  |  |  | 0% |  | - |  |  |
| **Diabetes** | | | | | | | | | | | | |
| 1 | randomised trials | no serious risk of bias | no serious inconsistency | no serious indirectness | no serious imprecision | none | - | - | RR 1.46 (1.15 to 1.98) | - | ⊕⊕⊕⊕ HIGH |  |
|  |  |  |  |  |  |  |  | 0% |  | - |  |  |

^1^ Wide credibility interval, including both no difference and important difference.
^2^ Wide credibility interval, including important differences in opposing directions.

Beta-blockers vs diuretics and/or beta-blockers

**Author(s): A Fretheim**
**Date:** 2011-05-31
**Question:** Should Beta-blockers vs diuretics and/or beta-blockers be used for hypertension?

| **Quality assessment** | | | | | | | **No of patients** | | **Effect** | | **Quality** | **Importance** |
| --- | --- | --- | --- | --- | --- | --- | --- | --- | --- | --- | --- | --- |
|  |  |  |  |  |  |  |  |  |  |  |  |  |
| **No of studies (direct comparisons)** | **Design** | **Risk of bias** | **Inconsistency** | **Indirectness** | **Imprecision** | **Other considerations** | **Beta-blockers** | **Diuretics and/or beta-blockers** | **Relative (95% CI)** | **Absolute** |  |  |
| **Total mortality** | | | | | | | | | | | | |
| 0 | only indirect comparisons |  |  |  | no serious imprecision | none | - | - | RR 1.19 (1.03 to 1.36) | - | ⊕⊕OO LOW |  |
|  |  |  |  |  |  |  |  | 0% |  | - |  |  |
| **Myocardial infarction** | | | | | | | | | | | | |
| 0 | only indirect comparisons |  |  |  | serious^1^ | none | - | - | RR 1.18 (0.95 to 1.48) | - | ⊕OOO VERY LOW |  |
|  |  |  |  |  |  |  |  | 0% |  | - |  |  |
| **Stroke** | | | | | | | | | | | | |
| 0 | only indirect comparisons |  |  |  | serious^1^ | none | - | - | RR 1.24 (0.95 to 1.58) | - | ⊕OOO VERY LOW |  |
|  |  |  |  |  |  |  |  | 0% |  | - |  |  |
| **Angina** | | | | | | | | | | | | |
| 0 | only indirect comparisons |  |  |  | very serious^2^ | none | - | - | RR 1.12 (0.18 to 4.24) | - | ⊕OOO VERY LOW |  |
|  |  |  |  |  |  |  |  | 0% |  | - |  |  |
| **Heart failure** | | | | | | | | | | | | |
| 0 | only indirect comparisons |  |  |  | serious^1^ | none | - | - | RR 1.17 (0.86 to 1.65) | - | ⊕OOO VERY LOW |  |
|  |  |  |  |  |  |  |  | 0% |  | - |  |  |
| **Diabetes** | | | | | | | | | | | | |
| 0 | only indirect comparisons |  |  |  | serious^1^ | none | - | - | RR 1.12 (0.81 to 1.64) | - | ⊕OOO VERY LOW |  |
|  |  |  |  |  |  |  |  | 0% |  | - |  |  |

^1^ Wide credibility interval, including both no difference and important difference.
^2^ Wide credibility interval, including important differences in opposite directions.

Beta-blockers vs "conventional drugs"

**Author(s): A Fretheim
Date: 2011-05-31
Question: Should Beta-blockers vs "conventional drugs" be used for hypertension?**

| **Quality assessment** | | | | | | | **No of patients** | | **Effect** | | **Quality** | **Importance** |
| --- | --- | --- | --- | --- | --- | --- | --- | --- | --- | --- | --- | --- |
|  |  |  |  |  |  |  |  |  |  |  |  |  |
| **No of studies (direct comparisons)** | **Design** | **Risk of bias** | **Inconsistency** | **Indirectness** | **Imprecision** | **Other considerations** | **Beta-blockers** | **"conventional drugs"** | **Relative (95% CI)** | **Absolute** |  |  |
| **Total mortality** | | | | | | | | | | | | |
| 0 | only indirect comparisons |  |  |  | very serious^1^ | none | - | - | RR 1.07 (0.28 to 4.69) | - | ⊕OOO VERY LOW |  |
|  |  |  |  |  |  |  |  | 0% |  | - |  |  |
| **Myocardial infarction** | | | | | | | | | | | | |
| 0 | only indirect comparisons |  |  |  | no serious imprecision | large effect^2^ | - | - | RR 0.45 (0.18 to 0.94) | - | ⊕⊕⊕O MODERATE |  |
|  |  |  |  |  |  |  |  | 0% |  | - |  |  |
| **Stroke** | | | | | | | | | | | | |
| 0 | only indirect comparisons |  |  |  | serious^3^ | none | - | - | RR 0.74 (0.46 to 1.15) | - | ⊕OOO VERY LOW |  |
|  |  |  |  |  |  |  |  | 0% |  | - |  |  |
| **Heart failure** | | | | | | | | | | | | |
| 0 | only indirect comparisons |  |  |  | very serious^1^ | none | - | - | RR 0.94 (0.54 to 1.56) | - | ⊕OOO VERY LOW |  |
|  |  |  |  |  |  |  |  | 0% |  | - |  |  |

^1^Wide credibility interval, including important differences in opposite directions..

^2^RR < 0.5

^3^Wide credibility interval, including both no difference and important difference

Beta-blockers vs placebo/control

**Author(s): A Fretheim**
**Date:** 2011-05-31
**Question:** Should Beta-blockers vs placebo/control be used for hypertension?

| **Quality assessment** | | | | | | | **No of patients** | | **Effect** | | **Quality** | **Importance** |
| --- | --- | --- | --- | --- | --- | --- | --- | --- | --- | --- | --- | --- |
|  |  |  |  |  |  |  |  |  |  |  |  |  |
| **No of studies (direct comparisons)** | **Design** | **Risk of bias** | **Inconsistency** | **Indirectness** | **Imprecision** | **Other considerations** | **Beta-blockers** | **Placebo/control** | **Relative (95% CI)** | **Absolute** |  |  |
| **Total mortality** | | | | | | | | | | | | |
| 2 | randomised trials | serious^1^ | no serious inconsistency | no serious indirectness | no serious imprecision | none | - | - | RR 0.97 (0.86 to 1.1) | - | ⊕⊕⊕O MODERATE |  |
|  |  |  |  |  |  |  |  | 0% |  | - |  |  |
| **Myocardial infarction** | | | | | | | | | | | | |
| 2 | randomised trials | serious^1^ | no serious inconsistency | no serious indirectness | no serious imprecision | none | - | - | RR 0.93 (0.77 to 1.13) | - | ⊕⊕⊕O MODERATE |  |
|  |  |  |  |  |  |  |  | 0% |  | - |  |  |
| **Stroke** | | | | | | | | | | | | |
| 2 | randomised trials | serious^1^ | no serious inconsistency | no serious indirectness | no serious imprecision | none | - | - | RR 0.73 (0.57 to 0.9) | - | ⊕⊕⊕O MODERATE |  |
|  |  |  |  |  |  |  |  | 0% |  | - |  |  |
| **Angina** | | | | | | | | | | | | |
| 0 | only indirect comparisons |  |  |  | Very serious^2^ | none | - | - | RR 1.52 (0.10 to 21.87) | - | ⊕OOO VERY LOW |  |
|  |  |  |  |  |  |  |  | 0% |  | - |  |  |
| **Heart failure** | | | | | | | | | | | | |
| 1 | randomised trials | Serious^3^ | no serious inconsistency | no serious indirectness | no serious imprecision | none | - | - | RR 0.63 (0.45 to 0.86) | - | ⊕⊕⊕O MODERATE |  |
|  |  |  |  |  |  |  |  | 0% |  | - |  |  |

^1^ Both studies rated "moderate quality" by expert group.

^2^Wide credibility interval, including important differences in opposite directions..

^3^ Study rated "moderate quality" by expert group.

ACE-inhibitors vs CCBs

**Author(s): A Fretheim**
**Date:** 2011-05-31
**Question:** Should ACE-inhibitors vs CCBs be used for hypertension?

| **Quality assessment** | | | | | | | **No of patients** | | **Effect** | | **Quality** | **Importance** |
| --- | --- | --- | --- | --- | --- | --- | --- | --- | --- | --- | --- | --- |
|  |  |  |  |  |  |  |  |  |  |  |  |  |
| **No of studies (direct comparisons)** | **Design** | **Risk of bias** | **Inconsistency** | **Indirectness** | **Imprecision** | **Other considerations** | **ACE-inhibitors** | **CCBs** | **Relative (95% CI)** | **Absolute** |  |  |
| **Total mortality** | | | | | | | | | | | | |
| 2 | randomised trials | serious^1^ | no serious inconsistency | no serious indirectness | no serious imprecision | none | - | - | RR 1.02 (0.95 to 1.1) | - | ⊕⊕⊕O MODERATE |  |
|  |  |  |  |  |  |  |  | 0% |  | - |  |  |
| **Myocardial infarction** | | | | | | | | | | | | |
| 2 | randomised trials | serious^1^ | no serious inconsistency | no serious indirectness | no serious imprecision | none | - | - | RR 0.96 (0.83 to 1.07) | - | ⊕⊕⊕O MODERATE |  |
|  |  |  |  |  |  |  |  | 0% |  | - |  |  |
| **Stroke** | | | | | | | | | | | | |
| 2 | randomised trials | serious^1^ | no serious inconsistency | no serious indirectness | no serious imprecision | none | - | - | RR 1.19 (1.03 to 1.38) | - | ⊕⊕⊕O MODERATE |  |
|  |  |  |  |  |  |  |  | 0% |  | - |  |  |
| **Angina** | | | | | | | | | | | | |
| 1 | randomised trials | serious^2^ | no serious inconsistency | no serious indirectness | very serious^3^ | none | - | - | RR 1.08 (0.48 to 2.44) | - | ⊕OOO VERY LOW |  |
|  |  |  |  |  |  |  |  | 0% |  | - |  |  |
| **Heart failiure** | | | | | | | | | | | | |
| 2 | randomised trials | serious^1^ | no serious inconsistency | no serious indirectness | no serious imprecision | none | - | - | RR 0.82 (0.69 to 0.94) | - | ⊕⊕⊕O MODERATE |  |
|  |  |  |  |  |  |  |  | 0% |  | - |  |  |
| **Diabetes** | | | | | | | | | | | | |
| 2 | randomised trials | serious^1^ | no serious inconsistency | no serious indirectness | serious^4^ | none | - | - | RR 0.89 (0.73 to 1.1) | - | ⊕⊕OO LOW |  |
|  |  |  |  |  |  |  |  | 0% |  | - |  |  |

^1^ Both trials rated "moderate quality" by expert group.
^2^ Trial rated "moderate quality" by expert group.
^3^ Wide credibility interval, including substantial differences in both directions.
^4^ Wide credibility interval, including both no difference and substantial difference.

ACE-inhibitors vs alpha-blockers

**Author(s): A Fretheim**
**Date:** 2011-05-31
**Question:** Should ACE-inhibitors vs alpha-blockers be used for hypertension?

| **Quality assessment** | | | | | | | **No of patients** | | **Effect** | | **Quality** | **Importance** |
| --- | --- | --- | --- | --- | --- | --- | --- | --- | --- | --- | --- | --- |
|  |  |  |  |  |  |  |  |  |  |  |  |  |
| **No of studies (direct comparisons)** | **Design** | **Risk of bias** | **Inconsistency** | **Indirectness** | **Imprecision** | **Other considerations** | **ACE-inhibitors** | **Alpha-blockers** | **Relative (95% CI)** | **Absolute** |  |  |
| **Total mortality** | | | | | | | | | | | | |
| 0 | only indirect comparisons |  |  |  | no serious imprecision | none | - | - | RR 0.98 (0.85 to 1.14) | - | ⊕⊕OO LOW |  |
|  |  |  |  |  |  |  |  | 0% |  | - |  |  |
| **Myocardial infarction** | | | | | | | | | | | | |
| 0 | only indirect comparisons |  |  |  | serious^1^ | none | - | - | RR 0.99 (0.77 to 1.27) | - | ⊕OOO VERY LOW |  |
|  |  |  |  |  |  |  |  | 0% |  | - |  |  |
| **Stroke** | | | | | | | | | | | | |
| 0 | only indirect comparisons |  |  |  | very serious^2^ | none | - | - | RR 0.91 (0.67 to 1.24) | - | ⊕OOO VERY LOW |  |
|  |  |  |  |  |  |  |  | 0% |  | - |  |  |
| **Angina** | | | | | | | | | | | | |
| 0 | only indirect comparisons |  |  |  | very serious^2^ | none | - | - | RR 0.91 (0.22 to 3.42) | - | ⊕OOO VERY LOW |  |
|  |  |  |  |  |  |  |  | 0% |  | - |  |  |
| **Heart failure** | | | | | | | | | | | | |
| 0 | only indirect comparisons |  |  |  | no serious imprecision | none | - | - | RR 0.58 (0.43 to 0.75) | - | ⊕⊕OO LOW |  |
|  |  |  |  |  |  |  |  | 0% |  | - |  |  |

^1^ Wide credibility interval, including both no difference and important difference.
^2^ Wide credibility interval, including important differences in opposite directions.

ACE-inhibitors vs ARBs

**Author(s): A Fretheim**
**Date:** 2011-05-31
**Question:** Should ACE-inhibitors vs ARBs be used for hypertension?

| **Quality assessment** | | | | | | | **No of patients** | | **Effect** | | **Quality** | **Importance** |
| --- | --- | --- | --- | --- | --- | --- | --- | --- | --- | --- | --- | --- |
|  |  |  |  |  |  |  |  |  |  |  |  |  |
| **No of studies (direct comparisons)** | **Design** | **Risk of bias** | **Inconsistency** | **Indirectness** | **Imprecision** | **Other considerations** | **ACE-inhibitors** | **ARBs** | **Relative (95% CI)** | **Absolute** |  |  |
| **Total mortality** | | | | | | | | | | | | |
| 0 | only indirect comparisons |  |  |  | no serious imprecision | none | - | - | RR 1.02 (0.91 to 1.14) | - | ⊕⊕OO LOW |  |
|  |  |  |  |  |  |  |  | 0% |  | - |  |  |
| **Myocardial infarction** | | | | | | | | | | | | |
| 0 | only indirect comparisons |  |  |  | serious^1^ | none | - | - | RR 0.84 (0.68 to 1.04) | - | ⊕OOO VERY LOW |  |
|  |  |  |  |  |  |  |  | 0% |  | - |  |  |
| **Stroke** | | | | | | | | | | | | |
| 0 | only indirect comparisons |  |  |  | serious^1^ | none | - | - | RR 1.08 (0.86 to 1.37) | - | ⊕OOO VERY LOW |  |
|  |  |  |  |  |  |  |  | 0% |  | - |  |  |
| **Angina** | | | | | | | | | | | | |
| 0 | only indirect comparisons |  |  |  | very serious^2^ | none | - | - | RR 0.86 (0.35 to 3.50) | - | ⊕OOO VERY LOW |  |
|  |  |  |  |  |  |  |  | 0% |  | - |  |  |
| **Heart failure** | | | | | | | | | | | | |
| 0 | only indirect comparisons |  |  |  | serious^1^ | none | - | - | RR 0.90 (0.67 to 1.1) | - | ⊕OOO VERY LOW |  |
|  |  |  |  |  |  |  |  | 0% |  | - |  |  |
| **Diabetes** | | | | | | | | | | | | |
| 0 | only indirect comparisons |  |  |  | serious^1^ | none | - | - | RR 1.11 (0.85 to 1.51) | - | ⊕OOO VERY LOW |  |
|  |  |  |  |  |  |  |  | 0% |  | - |  |  |

^1^ Wide credibility interval, including both no difference and important difference.
^2^ Wide credibility interval, including important differences in opposite directions.

ACE-inhibitors vs diuretics and/or beta-blockers

**Author(s): A Fretheim**
**Date:** 2011-05-31
**Question:** Should ACE-inhibitors vs diuretics and/or beta-blockers be used for hypertension?

| **Quality assessment** | | | | | | | **No of patients** | | **Effect** | | **Quality** | **Importance** |
| --- | --- | --- | --- | --- | --- | --- | --- | --- | --- | --- | --- | --- |
|  |  |  |  |  |  |  |  |  |  |  |  |  |
| **No of studies (direct comparisons)** | **Design** | **Risk of bias** | **Inconsistency** | **Indirectness** | **Imprecision** | **Other considerations** | **ACE-inhibitors** | **Diuretics and/or beta-blockers** | **Relative (95% CI)** | **Absolute** |  |  |
| **Total mortality** | | | | | | | | | | | | |
| 2 | randomised trials | serious^1^ | no serious inconsistency | no serious indirectness | no serious imprecision | none | - | - | RR 1.06 (0.97 to 1.16) | - | ⊕⊕⊕O MODERATE |  |
|  |  |  |  |  |  |  |  | 0% |  | - |  |  |
| **Myocardial infarction** | | | | | | | | | | | | |
| 2 | randomised trials | serious^1^ | no serious inconsistency | no serious indirectness | no serious imprecision | none | - | - | RR 0.97 (0.83 to 1.12) | - | ⊕⊕⊕O MODERATE |  |
|  |  |  |  |  |  |  |  | 0% |  | - |  |  |
| **Stroke** | | | | | | | | | | | | |
| 2 | randomised trials | serious^1^ | serious^2^ | no serious indirectness | serious^3^ | none | - | - | RR 1.10 (0.94 to 1.31) | - | ⊕OOO VERY LOW |  |
|  |  |  |  |  |  |  |  | 0% |  | - |  |  |
| **Angina** | | | | | | | | | | | | |
| 1 | randomised trials | serious^4^ | no serious inconsistency | no serious indirectness | very serious^5^ | none | - | - | RR 1.10 (0.47 to 2.55) | - | ⊕OOO VERY LOW |  |
|  |  |  |  |  |  |  |  | 0% |  | - |  |  |
| **Heart failure** | | | | | | | | | | | | |
| 2 | randomised trials | serious^1^ | no serious inconsistency | no serious indirectness | no serious imprecision | none | - | - | RR 0.96 (0.81 to 1.15) | - | ⊕⊕⊕O MODERATE |  |
|  |  |  |  |  |  |  |  | 0% |  | - |  |  |
| **Diabetes** | | | | | | | | | | | | |
| 2 | randomised trials | serious^1^ | no serious inconsistency | no serious indirectness | serious^3^ | none | - | - | RR 0.86 (0.7 to 1.06) | - | ⊕⊕OO LOW |  |
|  |  |  |  |  |  |  |  | 0% |  | - |  |  |

^1^ Both trials rated "moderate quality" by expert group.
^2^ I-squared = 83%
^3^ Wide credibility interval, including both no difference and substantial difference.
^4^ Trial rated "moderate quality" by expert group.
^5^ Wide credibility interval, including substantial differences in both directions.

ACE-inhibitors vs "conventional drugs"

**Author(s): A Fretheim**
**Date:** 2011-05-31
**Question:** Should ACE-inhibitors vs "conventional drugs" be used for hypertension?

| **Quality assessment** | | | | | | | **No of patients** | | **Effect** | | **Quality** | **Importance** |
| --- | --- | --- | --- | --- | --- | --- | --- | --- | --- | --- | --- | --- |
|  |  |  |  |  |  |  |  |  |  |  |  |  |
| **No of studies (direct comparisons)** | **Design** | **Risk of bias** | **Inconsistency** | **Indirectness** | **Imprecision** | **Other considerations** | **ACE-inhibitors** | **"conventional drugs"** | **Relative (95% CI)** | **Absolute** |  |  |
| **Total mortality** | | | | | | | | | | | | |
| 0 | only indirect comparisons |  |  |  | very serious^1^ | none | - | - | RR 0.95 (0.25 to 4.14) | - | ⊕OOO VERY LOW |  |
|  |  |  |  |  |  |  |  | 0% |  | - |  |  |
| **Myocardial infarction** | | | | | | | | | | | | |
| 0 | only indirect comparisons |  |  |  | no serious imprecision | large effect^2^ | - | - | RR 0.37 (0.15 to 0.77) | - | ⊕⊕⊕O MODERATE |  |
|  |  |  |  |  |  |  |  | 0% |  | - |  |  |
| **Stroke** | | | | | | | | | | | | |
| 0 | only indirect comparisons |  |  |  | serious^3^ | none | - | - | RR 0.65 (0.41 to 1.05) | - | ⊕OOO VERY LOW |  |
|  |  |  |  |  |  |  |  | 0% |  | - |  |  |
| **Heart failure** | | | | | | | | | | | | |
| 0 | only indirect comparisons |  |  |  | very serious^1^ | none | - | - | RR 0.78 (0.43 to 1.25) | - | ⊕OOO VERY LOW |  |
|  |  |  |  |  |  |  |  | 0% |  | - |  |  |

^1^Wide credibility interval, including important differences in opposite directions.

^2^RR < 0.5

^3^Wide credibility interval, including both no difference and important difference.

ACE-inhibitors vs placebo/control

**Author(s): A Fretheim**
**Date:** 2011-05-31
**Question:** Should ACE-inhibitors vs placebo/control be used for hypertension?

| **Quality assessment** | | | | | | | **No of patients** | | **Effect** | | **Quality** | **Importance** |
| --- | --- | --- | --- | --- | --- | --- | --- | --- | --- | --- | --- | --- |
|  |  |  |  |  |  |  |  |  |  |  |  |  |
| **No of studies (direct comparisons)** | **Design** | **Risk of bias** | **Inconsistency** | **Indirectness** | **Imprecision** | **Other considerations** | **ACE-inhibitors** | **Placebo/control** | **Relative (95% CI)** | **Absolute** |  |  |
| **Total mortality** | | | | | | | | | | | | |
| 1 | randomised trials | serious^1^ | no serious inconsistency | no serious indirectness | no serious imprecision | none | - | - | RR 0.87 (0.79 to 0.96) | - | ⊕⊕⊕O MODERATE |  |
|  |  |  |  |  |  |  |  | 0% |  | - |  |  |
| **Myocardial infarction** | | | | | | | | | | | | |
| 0 | only indirect comparisons |  |  |  | no serious imprecision | none | - | - | RR 0.76 (0.63 to 0.92) | - | ⊕⊕OO LOW |  |
|  |  |  |  |  |  |  |  | 0% |  | - |  |  |
| **Stroke** | | | | | | | | | | | | |
| 1 | randomised trials | serious^1^ | no serious inconsistency | no serious indirectness | no serious imprecision | none | - | - | RR 0.65 (0.53 to 0.78) | - | ⊕⊕⊕O MODERATE |  |
|  |  |  |  |  |  |  |  | 0% |  | - |  |  |
| **Angina** | | | | | | | | | | | | |
| 0 | only indirect comparisons |  |  |  | very serious^2^ | none | - | - | RR 1.57 (0.16 to 19.07) | - | ⊕OOO VERY LOW |  |
|  |  |  |  |  |  |  |  | 0% |  | - |  |  |
| **Heart failure** | | | | | | | | | | | | |
| 0 | only indirect comparisons |  |  |  | no serious imprecision | none | - | - | RR 0.51 (0.39 to 0.65) | - | ⊕⊕OO LOW |  |
|  |  |  |  |  |  |  |  | 0% |  | - |  |  |

^1^ Trial rate "moderate quality" by expert group.

^2^Wide credibility interval, including important differences in opposite directions.

CCBs vs alpha-blockers

**Author(s): A Fretheim**
**Date:** 2011-05-31
**Question:** Should CCBs vs alpha-blockers be used for hypertension?

| **Quality assessment** | | | | | | | **No of patients** | | **Effect** | | **Quality** | **Importance** |
| --- | --- | --- | --- | --- | --- | --- | --- | --- | --- | --- | --- | --- |
|  |  |  |  |  |  |  |  |  |  |  |  |  |
| **No of studies (direct comparisons)** | **Design** | **Risk of bias** | **Inconsistency** | **Indirectness** | **Imprecision** | **Other considerations** | **CCBs** | **Alpha-blockers** | **Relative (95% CI)** | **Absolute** |  |  |
| **Total mortality** | | | | | | | | | | | | |
| 0 | only indirect comparisons |  |  |  | no serious imprecision | none | - | - | RR 0.96 (0.83 to 1.11) | - | ⊕⊕OO LOW |  |
|  |  |  |  |  |  |  |  | 0% |  | - |  |  |
| **Myocardial infarction** | | | | | | | | | | | | |
| 0 | only indirect comparisons |  |  |  | serious^1^ | none | - | - | RR 1.03 (0.82 to 1.34) | - | ⊕OOO VERY LOW |  |
|  |  |  |  |  |  |  |  | 0% |  | - |  |  |
| **Stroke** | | | | | | | | | | | | |
| 0 | only indirect comparisons |  |  |  | serious^1^ | none | - | - | RR 0.77 (0.57 to 1.04) | - | ⊕OOO VERY LOW |  |
|  |  |  |  |  |  |  |  | 0% |  | - |  |  |
| **Angina** | | | | | | | | | | | | |
| 0 | only indirect comparisons |  |  |  | very serious^2^ | none | - | - | RR 0.85 (0.23 to 2.78) | - | ⊕OOO VERY LOW |  |
|  |  |  |  |  |  |  |  | 0% |  | - |  |  |
| **Heart failure** | | | | | | | | | | | | |
| 0 | only indirect comparisons |  |  |  | no serious imprecision | none | - | - | RR 0.70 (0.53 to 0.92) | - | ⊕⊕OO LOW |  |
|  |  |  |  |  |  |  |  | 0% |  | - |  |  |

^1^ Wide credibility interval, including both no difference and important difference.
^2^ Wide credibility interval, including important differences in opposite directions.

CCBs vs ARBs

**Author(s): A Fretheim**
**Date:** 2011-05-31
**Question:** Should CCBs vs ARBs be used for hypertension?

| **Quality assessment** | | | | | | | **No of patients** | | **Effect** | | **Quality** | **Importance** |
| --- | --- | --- | --- | --- | --- | --- | --- | --- | --- | --- | --- | --- |
|  |  |  |  |  |  |  |  |  |  |  |  |  |
| **No of studies (direct comparisons)** | **Design** | **Risk of bias** | **Inconsistency** | **Indirectness** | **Imprecision** | **Other considerations** | **CCBs** | **ARBs** | **Relative (95% CI)** | **Absolute** |  |  |
| **Total mortality** | | | | | | | | | | | | |
| 2 | randomised trials | no serious risk of bias | no serious inconsistency | no serious indirectness | no serious imprecision | none | - | - | RR 1.00 (0.91 to 1.1) | - | ⊕⊕⊕⊕ HIGH |  |
|  |  |  |  |  |  |  |  | 0% |  | - |  |  |
| **Myocardial infarction** | | | | | | | | | | | | |
| 2 | randomised trials | no serious risk of bias | no serious inconsistency | no serious indirectness | serious^1^ | none | - | - | RR 0.87 (0.74 to 1.06) | - | ⊕⊕⊕O MODERATE |  |
|  |  |  |  |  |  |  |  | 0% |  | - |  |  |
| **Stroke** | | | | | | | | | | | | |
| 2 | randomised trials | no serious risk of bias | no serious inconsistency | no serious indirectness | serious^1^ | none | - | - | RR 0.91 (0.75 to 1.11) | - | ⊕⊕⊕O MODERATE |  |
|  |  |  |  |  |  |  |  | 0% |  | - |  |  |
| **Angina** | | | | | | | | | | | | |
| 2 | randomised trials | no serious risk of bias | serious^2^ | no serious indirectness | serious^1^ | none | - | - | RR 0.81 (0.45 to 2.30) | - | ⊕⊕OO LOW |  |
|  |  |  |  |  |  |  |  | 0% |  | - |  |  |
| **Heart failure** | | | | | | | | | | | | |
| 2 | randomised trials | no serious risk of bias | no serious inconsistency | no serious indirectness | serious^1^ | none | - | - | RR 1.10 (0.87 to 1.31) | - | ⊕⊕⊕O MODERATE |  |
|  |  |  |  |  |  |  |  | 0% |  | - |  |  |
| **Diabetes** | | | | | | | | | | | | |
| 2 | randomised trials | no serious risk of bias | no serious inconsistency | no serious indirectness | no serious imprecision | none | - | - | RR 1.25 (1.02 to 1.56) | - | ⊕⊕⊕⊕ HIGH |  |
|  |  |  |  |  |  |  |  | 0% |  | - |  |  |

^1^ Wide credibility interval, including both no difference and substantial difference.
^2^ i-squared = 77%.

CCBs vs diuretics and/or beta-blockers

**Author(s): A Fretheim**
**Date:** 2011-05-31
**Question:** Should CCBs vs diuretics and/or beta-blockers be used for hypertension?

| **Quality assessment** | | | | | | | **No of patients** | | **Effect** | | **Quality** | **Importance** |
| --- | --- | --- | --- | --- | --- | --- | --- | --- | --- | --- | --- | --- |
|  |  |  |  |  |  |  |  |  |  |  |  |  |
| **No of studies (direct comparisons)** | **Design** | **Risk of bias** | **Inconsistency** | **Indirectness** | **Imprecision** | **Other considerations** | **CCBs** | **Diuretics and/or beta-blockers** | **Relative (95% CI)** | **Absolute** |  |  |
| **Total mortality** | | | | | | | | | | | | |
| 3 | randomised trials | serious^1^ | no serious inconsistency | no serious indirectness | no serious imprecision | none | - | - | RR 1.04 (0.95 to 1.13) | - | ⊕⊕⊕O MODERATE |  |
|  |  |  |  |  |  |  |  | 0% |  | - |  |  |
| **Myocardial infarction** | | | | | | | | | | | | |
| 3 | randomised trials | serious^1^ | serious^2^ | no serious indirectness | serious^3^ | none | - | - | RR 1.22 (0.89 to 6.63) | - | ⊕OOO VERY LOW |  |
|  |  |  |  |  |  |  |  | 0% |  | - |  |  |
| **Stroke** | | | | | | | | | | | | |
| 3 | randomised trials | serious^1^ | no serious inconsistency | no serious indirectness | no serious imprecision | none | - | - | RR 0.93 (0.81 to 1.08) | - | ⊕⊕⊕O MODERATE |  |
|  |  |  |  |  |  |  |  | 0% |  | - |  |  |
| **Angina** | | | | | | | | | | | | |
| 1 | randomised trials | no serious risk of bias | no serious inconsistency | no serious indirectness | very serious^4^ | none | - | - | RR 1.02 (0.43 to 2.37) | - | ⊕⊕OO LOW |  |
|  |  |  |  |  |  |  |  | 0% |  | - |  |  |
| **Heart failure** | | | | | | | | | | | | |
| 3 | randomised trials | serious^1^ | no serious inconsistency | no serious indirectness | no serious imprecision | none | - | - | RR 1.17 (1.01 to 1.4) | - | ⊕⊕⊕O MODERATE |  |
|  |  |  |  |  |  |  |  | 0% |  | - |  |  |
| **Diabetes** | | | | | | | | | | | | |
| 2 | randomised trials | serious^5^ | no serious inconsistency | no serious indirectness | no serious imprecision | none | - | - | RR 0.96 (0.78 to 1.19) | - | ⊕⊕⊕O MODERATE |  |
|  |  |  |  |  |  |  |  | 0% |  | - |  |  |

^1^ 2 of 3 trials rated "moderate quality" by expert group.
^2^ I-squared = 70%.
^3^ Wide credibility interval, including both no difference and substantial difference.
^4^ Wide credibility interval, including substantial difference in both directions.
^5^ Both trials rated "moderate quality" by expert group.

CCBs vs "conventional drugs"

**Author(s): A Fretheim**
**Date:** 2011-05-31
**Question:** Should CCBs vs "conventional drugs" be used for hypertension?

| **Quality assessment** | | | | | | | **No of patients** | | **Effect** | | **Quality** | **Importance** |
| --- | --- | --- | --- | --- | --- | --- | --- | --- | --- | --- | --- | --- |
|  |  |  |  |  |  |  |  |  |  |  |  |  |
| **No of studies (direct comparisons)** | **Design** | **Risk of bias** | **Inconsistency** | **Indirectness** | **Imprecision** | **Other considerations** | **CCBs** | **"conventional drugs"** | **Relative (95% CI)** | **Absolute** |  |  |
| **Total mortality** | | | | | | | | | | | | |
| 0 | only indirect comparisons |  |  |  | very serious^1^ | none | - | - | RR 0.93 (0.24 to 4.01) | - | ⊕OOO VERY LOW |  |
|  |  |  |  |  |  |  |  | 0% |  | - |  |  |
| **Myocardial infarction** | | | | | | | | | | | | |
| 0 | only indirect comparisons |  |  |  | no serious imprecision | large effect^2^ | - | - | RR 0.39 (0.16 to 0.8) | - | ⊕⊕⊕O MODERATE |  |
|  |  |  |  |  |  |  |  | 0% |  | - |  |  |
| **Stroke** | | | | | | | | | | | | |
| 0 | only indirect comparisons |  |  |  | no serious imprecision | none | - | - | RR 0.55 (0.35 to 0.87) | - | ⊕⊕OO LOW |  |
|  |  |  |  |  |  |  |  | 0% |  | - |  |  |
| **Heart failure** | | | | | | | | | | | | |
| 0 | only indirect comparisons |  |  |  | very serious^1^ | none | - | - | RR 0.95 (0.54 to 1.51) | - | ⊕OOO VERY LOW |  |
|  |  |  |  |  |  |  |  | 0% |  | - |  |  |

^1^ Wide credibility interval, including important differences in opposite directions.

^2^RR < 0.5

CCBs vs placebo/control

**Author(s): A Fretheim**
**Date:** 2011-05-31
**Question:** Should CCBs vs placebo/control be used for hypertension?

| **Quality assessment** | | | | | | | **No of patients** | | **Effect** | | **Quality** | **Importance** |
| --- | --- | --- | --- | --- | --- | --- | --- | --- | --- | --- | --- | --- |
|  |  |  |  |  |  |  |  |  |  |  |  |  |
| **No of studies (direct comparisons)** | **Design** | **Risk of bias** | **Inconsistency** | **Indirectness** | **Imprecision** | **Other considerations** | **CCBs** | **Placebo/control** | **Relative (95% CI)** | **Absolute** |  |  |
| **Total mortality** | | | | | | | | | | | | |
| 2 | randomised trials | no serious risk of bias | no serious inconsistency | no serious indirectness | no serious imprecision | none | - | - | RR 0.85 (0.78 to 0.93) | - | ⊕⊕⊕⊕ HIGH |  |
|  |  |  |  |  |  |  |  | 0% |  | - |  |  |
| **Myocardial infarction** | | | | | | | | | | | | |
| 1 | randomised trials | no serious risk of bias | no serious inconsistency | no serious indirectness | no serious imprecision | none | - | - | RR 0.80 (0.67 to 0.95) | - | ⊕⊕⊕⊕ HIGH |  |
|  |  |  |  |  |  |  |  | 0% |  | - |  |  |
| **Stroke** | | | | | | | | | | | | |
| 2 | randomised trials | no serious risk of bias | no serious inconsistency | no serious indirectness | no serious imprecision | none | - | - | RR 0.55 (0.46 to 0.64) | - | ⊕⊕⊕⊕ HIGH |  |
|  |  |  |  |  |  |  |  | 0% |  | - |  |  |
| **Angina** | | | | | | | | | | | | |
| 0 | only indirect comparisons |  |  |  | veryserious^1^ | none | - | - | RR 1.45 (0.16 to 16.44) | - | ⊕OOO VERY LOW |  |
|  |  |  |  |  |  |  |  | 0% |  | - |  |  |
| **Heart failure** | | | | | | | | | | | | |
| 1 | randomised trials | no serious risk of bias | no serious inconsistency | no serious indirectness | no serious imprecision | none | - | - | RR 0.63 (0.49 to 0.78) | - | ⊕⊕⊕⊕ HIGH |  |
|  |  |  |  |  |  |  |  | 0% |  | - |  |  |

^1^ Wide credibility interval, including important differences in opposite directions.

Alpha-blockers vs ARBs

**Author(s): A Fretheim**
**Date:** 2011-05-31
**Question:** Should Alpha-blockers vs ARBs be used for hypertension?

| **Quality assessment** | | | | | | | **No of patients** | | **Effect** | | **Quality** | **Importance** |
| --- | --- | --- | --- | --- | --- | --- | --- | --- | --- | --- | --- | --- |
|  |  |  |  |  |  |  |  |  |  |  |  |  |
| **No of studies (direct comparisons)** | **Design** | **Risk of bias** | **Inconsistency** | **Indirectness** | **Imprecision** | **Other considerations** | **Alpha-blockers** | **ARBs** | **Relative (95% CI)** | **Absolute** |  |  |
| **Total mortality** | | | | | | | | | | | | |
| 0 | only indirect comparisons |  |  |  | no serious imprecision | none | - | - | RR 1.04 (0.88 to 1.23) | - | ⊕⊕OO LOW |  |
|  |  |  |  |  |  |  |  | 0% |  | - |  |  |
| **Myocardial infarction** | | | | | | | | | | | | |
| 0 | only indirect comparisons |  |  |  | serious^1^ | none | - | - | RR 0.84 (0.63 to 1.14) | - | ⊕OOO VERY LOW |  |
|  |  |  |  |  |  |  |  | 0% |  | - |  |  |
| **Stroke** | | | | | | | | | | | | |
| 0 | only indirect comparisons |  |  |  | serious^1^ | none | - | - | RR 1.20 (0.85 to 1.69) | - | ⊕OOO VERY LOW |  |
|  |  |  |  |  |  |  |  | 0% |  | - |  |  |
| **Angina** | | | | | | | | | | | | |
| 0 | only indirect comparisons |  |  |  | very serious^2^ | none | - | - | RR 0.95 (0.29 to 5.71) | - | ⊕OOO VERY LOW |  |
|  |  |  |  |  |  |  |  | 0% |  | - |  |  |
| **Heart failure** | | | | | | | | | | | | |
| 0 | only indirect comparisons |  |  |  | no serious imprecision | none | - | - | RR 1.57 (1.09 to 2.12) | - | ⊕⊕OO LOW |  |
|  |  |  |  |  |  |  |  | 0% |  | - |  |  |

^1^ Wide credibility interval, including both no difference and important difference.
^2^ Wide credibility interval, including important differences in opposite directions.

Alpha-blockers vs diuretics and/or beta-blockers

**Author(s): A Fretheim**
**Date:** 2011-05-31
**Question:** Should Alpha-blockers vs diuretics and/or beta-blockers be used for hypertension?

| **Quality assessment** | | | | | | | **No of patients** | | **Effect** | | **Quality** | **Importance** |
| --- | --- | --- | --- | --- | --- | --- | --- | --- | --- | --- | --- | --- |
|  |  |  |  |  |  |  |  |  |  |  |  |  |
| **No of studies (direct comparisons)** | **Design** | **Risk of bias** | **Inconsistency** | **Indirectness** | **Imprecision** | **Other considerations** | **Alpha-blockers** | **Diuretics and/or beta-blockers** | **Relative (95% CI)** | **Absolute** |  |  |
| **Total mortality** | | | | | | | | | | | | |
| 0 | only indirect comparisons |  |  |  | serious^1^ | none | - | - | RR 1.09 (0.92 to 1.27) | - | ⊕OOO VERY LOW |  |
|  |  |  |  |  |  |  |  | 0% |  | - |  |  |
| **Myocardial infarction** | | | | | | | | | | | | |
| 0 | only indirect comparisons |  |  |  | serious^1^ | none | - | - | RR 0.98 (0.74 to 1.28) | - | ⊕OOO VERY LOW |  |
|  |  |  |  |  |  |  |  | 0% |  | - |  |  |
| **Stroke** | | | | | | | | | | | | |
| 0 | only indirect comparisons |  |  |  | serious^1^ | none | - | - | RR 1.21 (0.88 to 1.68) | - | ⊕OOO VERY LOW |  |
|  |  |  |  |  |  |  |  | 0% |  | - |  |  |
| **Angina** | | | | | | | | | | | | |
| 0 | only indirect comparisons |  |  |  | very serious^2^ | none | - | - | RR 1.20 (0.30 to 5.46) | - | ⊕OOO VERY LOW |  |
|  |  |  |  |  |  |  |  | 0% |  | - |  |  |
| **Heart failure** | | | | | | | | | | | | |
| 0 | only indirect comparisons |  |  |  | no serious imprecision | none | - | - | RR 1.67 (1.26 to 2.31) | - | ⊕⊕OO LOW |  |
|  |  |  |  |  |  |  |  | 0% |  | - |  |  |

^1^ Wide credibility interval, including both no difference and important difference.
^2^ Wide credibility interval, including important differences in opposite directions.

Alpha-blockers vs "conventional drugs"

**Author(s): A Fretheim**
**Date:** 2011-05-31
**Question:** Should Alpha-blockers vs "conventional drugs" be used for hypertension?

| **Quality assessment** | | | | | | | **No of patients** | | **Effect** | | **Quality** | **Importance** |
| --- | --- | --- | --- | --- | --- | --- | --- | --- | --- | --- | --- | --- |
|  |  |  |  |  |  |  |  |  |  |  |  |  |
| **No of studies (direct comparisons)** | **Design** | **Risk of bias** | **Inconsistency** | **Indirectness** | **Imprecision** | **Other considerations** | **Alpha-blockers** | **"conventional drugs"** | **Relative (95% CI)** | **Absolute** |  |  |
| **Total mortality** | | | | | | | | | | | | |
| 0 | only indirect comparisons |  |  |  | very serious^1^ | none | - | - | RR 0.97 (0.25 to 4.23) | - | ⊕OOO VERY LOW |  |
|  |  |  |  |  |  |  |  | 0% |  | - |  |  |
| **Myocardial infarction** | | | | | | | | | | | | |
| 0 | only indirect comparisons |  |  |  | no serious imprecision | large effect^2^ | - | - | RR 0.38 (0.15 to 0.79) | - | ⊕⊕⊕O MODERATE |  |
|  |  |  |  |  |  |  |  | 0% |  | - |  |  |
| **Stroke** | | | | | | | | | | | | |
| 0 | only indirect comparisons |  |  |  | serious^3^ | none | - | - | RR 0.73 (0.43 to 1.23) | - | ⊕OOO VERY LOW |  |
|  |  |  |  |  |  |  |  | 0% |  | - |  |  |
| **Heart failure** | | | | | | | | | | | | |
| 0 | only indirect comparisons |  |  |  | very serious^1^ | none | - | - | RR 1.36 (0.72 to 2.32) | - | ⊕OOO VERY LOW |  |
|  |  |  |  |  |  |  |  | 0% |  | - |  |  |

^1^ Wide credibility interval, including important differences in opposite directions.

^2^ RR < 0.5

^3^ Wide credibility interval, including both no difference and important difference.

Alpha-blockers vs placebo/control

**Author(s): A Fretheim**
**Date:** 2011-05-31
**Question:** Should Alpha-blockers vs placebo/control be used for hypertension?

| **Quality assessment** | | | | | | | **No of patients** | | **Effect** | | **Quality** | **Importance** |
| --- | --- | --- | --- | --- | --- | --- | --- | --- | --- | --- | --- | --- |
|  |  |  |  |  |  |  |  |  |  |  |  |  |
| **No of studies (direct comparisons)** | **Design** | **Risk of bias** | **Inconsistency** | **Indirectness** | **Imprecision** | **Other considerations** | **Alpha-blockers** | **Placebo/control** | **Relative (95% CI)** | **Absolute** |  |  |
| **Total mortality** | | | | | | | | | | | | |
| 0 | only indirect comparisons |  |  |  | no serious imprecision | none | - | - | RR 0.89 (0.77 to 1.03) | - | ⊕⊕OO LOW |  |
|  |  |  |  |  |  |  |  | 0% |  | - |  |  |
| **Myocardial infarction** | | | | | | | | | | | | |
| 0 | only indirect comparisons |  |  |  | no serious imprecision | none | - | - | RR 0.82 (0.59 to 1) | - | ⊕⊕OO LOW |  |
|  |  |  |  |  |  |  |  | 0% |  | - |  |  |
| **Stroke** | | | | | | | | | | | | |
| 0 | only indirect comparisons |  |  |  | no serious imprecision | none | - | - | RR 0.72 (0.52 to 0.96) | - | ⊕⊕OO LOW |  |
|  |  |  |  |  |  |  |  | 0% |  | - |  |  |
| **Angina** | | | | | | | | | | | | |
| 0 | only indirect comparisons |  |  |  | very serious^1^ | none | - | - | RR 1.77 (0.17 to 22.73) | - | ⊕OOO VERY LOW |  |
|  |  |  |  |  |  |  |  | 0% |  | - |  |  |
| **Heart failure** | | | | | | | | | | | | |
| 0 | only indirect comparisons |  |  |  | serious^2^ | none | - | - | RR 0.90 (0.64 to 1.21) | - | ⊕OOO VERY LOW |  |
|  |  |  |  |  |  |  |  | 0% |  | - |  |  |

^1^ Wide credibility interval, including both no difference and important difference.
^2^ Wide credibility interval, including important differences in opposite directions.

ARBs vs diuretics and/or beta-blockers

**Author(s): A Fretheim**
**Date:** 2011-05-31
**Question:** Should ARBs vs diuretics and/or beta-blockers be used for hypertension?

| **Quality assessment** | | | | | | | **No of patients** | | **Effect** | | **Quality** | **Importance** |
| --- | --- | --- | --- | --- | --- | --- | --- | --- | --- | --- | --- | --- |
|  |  |  |  |  |  |  |  |  |  |  |  |  |
| **No of studies (direct comparisons)** | **Design** | **Risk of bias** | **Inconsistency** | **Indirectness** | **Imprecision** | **Other considerations** | **ARBs** | **Diuretics and/or beta-blockers** | **Relative (95% CI)** | **Absolute** |  |  |
| **Total mortality** | | | | | | | | | | | | |
| 0 | only indirect comparisons |  |  |  | no serious imprecision | none | - | - | RR 1.22 (1.09 to 1.37) | - | ⊕⊕OO LOW |  |
|  |  |  |  |  |  |  |  | 0% |  | - |  |  |
| **Myocardial infarction** | | | | | | | | | | | | |
| 0 | only indirect comparisons |  |  |  | Serious^1^ | none | - | - | RR 1.16 (0.92 to 1.43) | - | ⊕OOO VERY LOW |  |
|  |  |  |  |  |  |  |  | 0% |  | - |  |  |
| **Stroke** | | | | | | | | | | | | |
| 0 | only indirect comparisons |  |  |  | serious^1^ | none | - | - | RR 1.02 (0.8 to 1.29) | - | ⊕OOO VERY LOW |  |
|  |  |  |  |  |  |  |  | 0% |  | - |  |  |
| **Angina** | | | | | | | | | | | | |
| 0 | only indirect comparisons |  |  |  | very serious^2^ | none | - | - | RR 1.27 (0.30 to 3.20) | - | ⊕OOO VERY LOW |  |
|  |  |  |  |  |  |  |  | 0% |  | - |  |  |
| **Heart failure** | | | | | | | | | | | | |
| 0 | only indirect comparisons |  |  |  | serious^1^ | none | - | - | RR 1.07 (0.86 to 1.45) | - | ⊕OOO VERY LOW |  |
|  |  |  |  |  |  |  |  | 0% |  | - |  |  |
| **Diabetes** | | | | | | | | | | | | |
| 0 | only indirect comparisons |  |  |  | serious^1^ | none | - | - | RR 0.77 (0.57 to 1.03) | - | ⊕OOO VERY LOW |  |
|  |  |  |  |  |  |  |  | 0% |  | - |  |  |

^1^ Wide credibility interval, including both no difference and important difference.
^2^ Wide credibility interval, including important differences in opposite directions.

ARBs vs "conventional drugs"

**Author(s): A Fretheim**
**Date:** 2011-05-31
**Question:** Should ARBs vs "conventional drugs" be used for hypertension?

| **Quality assessment** | | | | | | | **No of patients** | | **Effect** | | **Quality** | **Importance** |
| --- | --- | --- | --- | --- | --- | --- | --- | --- | --- | --- | --- | --- |
|  |  |  |  |  |  |  |  |  |  |  |  |  |
| **No of studies (direct comparisons)** | **Design** | **Risk of bias** | **Inconsistency** | **Indirectness** | **Imprecision** | **Other considerations** | **ARBs** | **"conventional drugs"** | **Relative (95% CI)** | **Absolute** |  |  |
| **Total mortality** | | | | | | | | | | | | |
| 1 | randomised trials | serious^1^ | no serious inconsistency | no serious indirectness | very serious^2^ | none | - | - | RR 0.94 (0.24 to 4.06) | - | ⊕OOO VERY LOW |  |
|  |  |  |  |  |  |  |  | 0% |  | - |  |  |
| **Myocardial infarction** | | | | | | | | | | | | |
| 1 | randomised trials | serious^1^ | no serious inconsistency | no serious indirectness | no serious imprecision | none | - | - | RR 0.45 (0.18 to 0.82) | - | ⊕⊕⊕O MODERATE |  |
|  |  |  |  |  |  |  |  | 0% |  | - |  |  |
| **Stroke** | | | | | | | | | | | | |
| 1 | randomised trials | serious^1^ | no serious inconsistency | no serious indirectness | no serious imprecision | none | - | - | RR 0.60 (0.4 to 0.9) | - | ⊕⊕⊕O MODERATE |  |
|  |  |  |  |  |  |  |  | 0% |  | - |  |  |
| **Heart failure** | | | | | | | | | | | | |
| 1 | randomised trials | serious^1^ | no serious inconsistency | no serious indirectness | very serious^2^ | none | - | - | RR 0.86 (0.52 to 1.36) | - | ⊕OOO VERY LOW |  |
|  |  |  |  |  |  |  |  | 0% |  | - |  |  |

^1^ Trial rated "moderate qualilty" by exper group.
^2^ Wide credibility interval, including substantial differences in both directions.

ARBs vs placebo/control

**Author(s): A Fretheim**
**Date:** 2011-05-31
**Question:** Should ARBs vs placebo/control be used for hypertension?

| **Quality assessment** | | | | | | | **No of patients** | | **Effect** | | **Quality** | **Importance** |
| --- | --- | --- | --- | --- | --- | --- | --- | --- | --- | --- | --- | --- |
|  |  |  |  |  |  |  |  |  |  |  |  |  |
| **No of studies (direct comparisons)** | **Design** | **Risk of bias** | **Inconsistency** | **Indirectness** | **Imprecision** | **Other considerations** | **ARBs** | **Placebo/control** | **Relative (95% CI)** | **Absolute** |  |  |
| **Total mortality** | | | | | | | | | | | | |
| 0 | only indirect comparisons |  |  |  | no serious imprecision | none | - | - | RR 0.85 (0.76 to 0.96) | - | ⊕⊕OO LOW |  |
|  |  |  |  |  |  |  |  | 0% |  | - |  |  |
| **Myocardial infarction** | | | | | | | | | | | | |
| 0 | only indirect comparisons |  |  |  | serious^1^ | none | - | - | RR 0.91 (0.72 to 1.14) | - | ⊕OOO VERY LOW |  |
|  |  |  |  |  |  |  |  | 0% |  | - |  |  |
| **Stroke** | | | | | | | | | | | | |
| 0 | only indirect comparisons |  |  |  | no serious imprecision | none | - | - | RR 0.60 (0.47 to 0.75) | - | ⊕⊕OO LOW |  |
|  |  |  |  |  |  |  |  | 0% |  | - |  |  |
| **Angina** | | | | | | | | | | | | |
| 0 | only indirect comparisons |  |  |  | very serious^2^ | none | - | - | RR 1.70 (0.15 to 20.46) | - | ⊕OOO VERY LOW |  |
|  |  |  |  |  |  |  |  | 0% |  | - |  |  |
| **Heart failure** | | | | | | | | | | | | |
| 0 | only indirect comparisons |  |  |  | no serious imprecision | none | - | - | RR 0.57 (0.44 to 0.77) | - | ⊕⊕OO LOW |  |
|  |  |  |  |  |  |  |  | 0% |  | - |  |  |

^1^ Wide credibility interval, including both no difference and important difference.
^2^ Wide credibility interval, including important differences in opposite directions.

Diuretics and/or beta-blockers vs "conventional drugs"

**Author(s): A Fretheim**
**Date:** 2011-05-31
**Question:** Should Diuretics and/or beta-blockers vs "conventional drugs" be used for hypertension?

| **Quality assessment** | | | | | | | **No of patients** | | **Effect** | | **Quality** | **Importance** |
| --- | --- | --- | --- | --- | --- | --- | --- | --- | --- | --- | --- | --- |
|  |  |  |  |  |  |  |  |  |  |  |  |  |
| **No of studies (direct comparisons)** | **Design** | **Risk of bias** | **Inconsistency** | **Indirectness** | **Imprecision** | **Other considerations** | **Diuretics and/or beta-blockers** | **"conventional drugs"** | **Relative (95% CI)** | **Absolute** |  |  |
| **Total mortality** | | | | | | | | | | | | |
| 0 | only indirect comparisons |  |  |  | very serious^1^ | none | - | - | RR 0,90 (0.23 to 3.92) | - | ⊕OOO VERY LOW |  |
|  |  |  |  |  |  |  |  | 0% |  | - |  |  |
| **Myocardial infarction** | | | | | | | | | | | | |
| 0 | only indirect comparisons |  |  |  | no serious imprecision | large effect^2^ | - | - | RR 0.39 (0.16 to 0.8) | - | ⊕⊕⊕O MODERATE |  |
|  |  |  |  |  |  |  |  | 0% |  | - |  |  |
| **Stroke** | | | | | | | | | | | | |
| 0 | only indirect comparisons |  |  |  | no serious imprecision | none | - | - | RR 0.59 (0.37 to 0.95) | - | ⊕⊕OO LOW |  |
|  |  |  |  |  |  |  |  | 0% |  | - |  |  |
| **Heart failure** | | | | | | | | | | | | |
| 0 | only indirect comparisons |  |  |  | very serious^1^ | none | - | - | RR 0.81 (0.44 to 1.31) | - | ⊕OOO VERY LOW |  |
|  |  |  |  |  |  |  |  | 0% |  | - |  |  |

^1^ Wide credibility interval, including important differences in opposite directions.

^2^ RR < 0.5

Diuretics and/or beta-blockers vs placebo/control

**Author(s): A Fretheim**
**Date:** 2011-05-31
**Question:** Should Diuretics and/or beta-blockers vs placebo/control be used for hypertension?

| **Quality assessment** | | | | | | | **No of patients** | | **Effect** | | **Quality** | **Importance** |
| --- | --- | --- | --- | --- | --- | --- | --- | --- | --- | --- | --- | --- |
|  |  |  |  |  |  |  |  |  |  |  |  |  |
| **No of studies (direct comparisons)** | **Design** | **Risk of bias** | **Inconsistency** | **Indirectness** | **Imprecision** | **Other considerations** | **Diuretics and/or beta-blockers** | **Placebo/control** | **Relative (95% CI)** | **Absolute** |  |  |
| **Total mortality** | | | | | | | | | | | | |
| 1 | randomised trials | no serious risk of bias | no serious inconsistency | no serious indirectness | no serious imprecision | none | - | - | RR 0.82 (0.73 to 0.92) | - | ⊕⊕⊕⊕ HIGH |  |
|  |  |  |  |  |  |  |  | 0% |  | - |  |  |
| **Myocardial infarction** | | | | | | | | | | | | |
| 1 | randomised trials | no serious risk of bias | no serious inconsistency | no serious indirectness | no serious imprecision | none | - | - | RR 0.79 (0.64 to 0.97) | - | ⊕⊕⊕⊕ HIGH |  |
|  |  |  |  |  |  |  |  | 0% |  | - |  |  |
| **Stroke** | | | | | | | | | | | | |
| 1 | randomised trials | no serious risk of bias | no serious inconsistency | no serious indirectness | no serious imprecision | none | - | - | RR 0.59 (0.48 to 0.72) | - | ⊕⊕⊕⊕ HIGH |  |
|  |  |  |  |  |  |  |  | 0% |  | - |  |  |
| **Angina** | | | | | | | | | | | | |
| 0 | only indirect comparisons |  |  |  | Very serious^1^ | none | - | - | RR 1.43 (0.14 to 18.07) | - | ⊕OOO VERY LOW |  |
|  |  |  |  |  |  |  |  | 0% |  | - |  |  |
| **Heart failure** | | | | | | | | | | | | |
| 0 | only indirect comparisons |  |  |  | no serious imprecision | none | - | - | RR 0.53 (0.4 to 0.68) | - | ⊕⊕OO LOW |  |
|  |  |  |  |  |  |  |  | 0% |  | - |  |  |

^1^ Wide credibility interval, including important differences in opposite directions.

"Conventional drugs" vs placebo/control

**Author(s): A Fretheim**
**Date:** 2011-05-31
**Question:** Should "Conventional drugs" vs placebo/control be used for hypertension?

| **Quality assessment** | | | | | | | **No of patients** | | **Effect** | | **Quality** | **Importance** |
| --- | --- | --- | --- | --- | --- | --- | --- | --- | --- | --- | --- | --- |
|  |  |  |  |  |  |  |  |  |  |  |  |  |
| **No of studies (direct comparisons)** | **Design** | **Risk of bias** | **Inconsistency** | **Indirectness** | **Imprecision** | **Other considerations** | **"Conventional drugs"** | **Placebo/control** | **Relative (95% CI)** | **Absolute** |  |  |
| **Total mortality** | | | | | | | | | | | | |
| 0 | only indirect comparisons |  |  |  | very serious^1^ | none | - | - | RR 0.91 (0.25 to 3.45) | - | ⊕OOO VERY LOW |  |
|  |  |  |  |  |  |  |  | 0% |  | - |  |  |
| **Myocardial infarction** | | | | | | | | | | | | |
| 0 | only indirect comparisons |  |  |  | no serious imprecision | large effect^2^ | - | - | RR 2.04 (1 to 5.13) | - | ⊕⊕⊕O MODERATE |  |
|  |  |  |  |  |  |  |  | 0% |  | - |  |  |
| **Stroke** | | | | | | | | | | | | |
| 0 | only indirect comparisons |  |  |  | very serious^1^ | none | - | - | RR 1.00 (0.62 to 1.57) | - | ⊕OOO VERY LOW |  |
|  |  |  |  |  |  |  |  | 0% |  | - |  |  |
| **Heart failure** | | | | | | | | | | | | |
| 0 | only indirect comparisons |  |  |  | serious^3^ | none | - | - | RR 0.66 (0.39 to 1.18) | - | ⊕OOO VERY LOW |  |
|  |  |  |  |  |  |  |  | 0% |  | - |  |  |

^1^ Wide credibility interval, including important differences in opposite directions.

^2^ RR > 2.0

^3^ Wide credibility interval including both no difference and important difference.
